# Combination immunotherapy in hepatocellular carcinoma: synergies among immune checkpoints, TKIs, and chemotherapy

**DOI:** 10.1186/s13045-025-01739-6

**Published:** 2025-09-26

**Authors:** Suoyi Dai, Yuhang Chen, Wenxun Cai, Shu Dong, Jiangang Zhao, Lianyu Chen, Chien-Shan Cheng

**Affiliations:** 1 Department of Integrative Oncology, Fudan University Shanghai Cancer Center, Shanghai, 200032 China; 2 Department of Oncology, Shanghai Medical College, Fudan University, 270 Dong-An Road, Shanghai, 200032 China; 3 Department of Oncology, Shaoxing Central Hospital, Shaoxing, 312030 China; 4 Department of Oncology, The Central Affiliated Hospital, Shaoxing University, Shaoxing, 312030 China

**Keywords:** Immune checkpoint inhibitors (ICIs), Tyrosine kinase inhibitors (TKIs), Chemotherapy, Combination immunotherapy, Hepatocellular carcinoma (HCC)

## Abstract

Combination therapy is rapidly becoming the cornerstone of hepatocellular carcinoma (HCC) treatment. Immune checkpoint inhibitors (ICIs) have emerged as a central strategy in systemic therapy, yet their efficacy as monotherapies remains limited. Consequently, combinatorial approaches, such as ICIs-Tyrosine kinase inhibitors (TKIs), ICIs-chemotherapy, and dual ICI regimens, are gaining momentum. While clinical trials have established efficacy benchmarks, mechanistic insights remain scarce, partly due to the limitations of current preclinical models in mimicking the complex tumor microenvironment (TME). Given the substantial heterogeneity of HCC, spanning genetic, transcriptomic, and immunologic dimensions, treatment outcomes vary widely. Additional factors such as gut microbiota and epigenetic modifications further influence therapeutic response and resistance. Although PD-1, PD-L1, and CTLA-4 inhibitors are widely used, unresponsiveness is common. Novel targets such as LAG-3, TIM-3, TIGIT, and VISTA, as well as strategies to reprogram fibrotic and immunosuppressive TME, are under active investigation. Ultimately, translating basic insights into personalized therapy will depend on predictive biomarkers and integrated analyses that account for the complex interactions among tumor cells, the immune system, and the TME. This review synthesizes current knowledge and cellular mechanisms underpinning combination therapies, highlights therapeutic synergies, and discusses emerging directions for stratified treatment in HCC.

## Background

Hepatocellular carcinoma (HCC) accounts for roughly 90% of all primary liver cancers and remains one of the leading causes of cancer-related mortality worldwide. HCC arises from diverse etiologies, including chronic hepatitis B virus (HBV) and hepatitis C virus (HCV) infections, metabolic dysfunction-associated steatotic liver disease (MASLD; formerly known as non-alcoholic fatty liver disease, NAFLD), metabolic dysfunction-associated steatohepatitis (MASH; formerly known as non-alcoholic steatohepatitis, NASH), and alcohol-related liver disease [1]. Prognosis of HCC remains poor, with a 5-year survival rate below 20%, primarily because most patients are diagnosed at advanced stages when curative interventions, such as resection or liver transplantation, are no longer feasible [2]. 

Multikinase inhibitor sorafenib marks the beginning of systemic treatment for unresectable HCC. Its pivotal role was established through the SHARP trial (NCT00105443), a landmark phase III study demonstrating a significant improvement in median overall survival (OS) (10.7 months with sorafenib vs. 7.9 months with placebo) and time to radiologic progression (5.5 vs. 2.8 months) in patients with advanced HCC[3]. These findings led to regulatory approvals worldwide, positioning sorafenib as the first systemic therapy to offer a survival benefit in this setting. Despite its groundbreaking impact, sorafenib's clinical benefits were modest, with low objective response rates (ORRs) and the eventual development of resistance.

Beyond sorafenib, recent therapeutic advances, particularly immune checkpoint inhibitors (ICIs), tyrosine kinase inhibitors (TKIs), and combination regimens, have reshaped the treatment paradigm (Table 1). However, most patients fail to achieve sustained clinical benefit with current systemic therapies. Single ICI monoclonal antibody (mAb) achieves modest response rates (approximately 15%−20%), and TKIs offer limited survival benefits [4–7]. Moreover, recurrence is common even after curative-intent treatments, underscoring the limitations of current approaches and the urgent need for more effective systemic strategies [8, 9].

**Table 1:** Approved systemic therapies and combination regimens for HCC

Agents/Regimens	Brand Name	Approval	Date	Line of Therapy	Type	Supporting Study
Sorafenib	Nexavar	FDA	2007	First-line	TKI	SHARP
Lenvatinib	Lenvima	FDA	2018	First-line	TKI	REFLECT
Atezolizumab + Bevacizumab	Tecentriq + Avastin	FDA	2020	First-line	PD-L1 inhibitor + VEGF inhibitor	IMbrave150
Donafenib	Zepsun	NMPA	2021	First-line	TKI	ZGDH3
Sintilimab + IBI305	Tyvyt + Byvasda	NMPA	2021	First-line	PD-1 inhibitor + VEGF inhibitor	ORIENT-32
Durvalumab + Tremelimumab	Imfinzi + Imjudo	FDA	2022	First-line	PD-L1 inhibitor + CTLA-4 inhibitor	HIMALAYA
Camrelizumab + Rivoceranib	AiRuiKa + Apatinib	NMPA	2023	First-line	PD-1 inhibitor + VEGFR-2 inhibitor	CARES-310
Regorafenib	Stivarga	FDA	2017	Second-line	TKI	RESORCE
Pembrolizumab	Keytruda	FDA	2018	Second-line	PD-1 inhibitor	KEYNOTE-224
Cabozantinib	Cabometyx	FDA	2019	Second-line	TKI	CELESTIAL
Ramucirumab	Cyramza	FDA	2019	Second-line	VEGFR-2 inhibitor	REACH 2
Nivolumab + Ipilimumab	Opdivo + Yervoy	FDA	2020	Second-line	PD-1 inhibitor + CTLA-4 inhibitor	CHECKMATE-040

*FDA* Food and Drug Administration, *NMPA* National Medical Products Administration

This limited efficacy is largely attributed to the unique immunosuppressive tumor microenvironment (TME) of HCC, which hampers effective immune responses and facilitates tumor progression. The upregulation of vascular endothelial growth factor (VEGF) contributes to abnormal vasculature and immune exclusion, limiting immune cell infiltration and reducing the efficacy of ICIs [10]. TKIs, such as lenvatinib and cabozantinib, can normalize tumor vasculature and enhance T-cell infiltration, thereby offering mechanistic synergy with ICIs [11]. Likewise, certain chemotherapeutic agents can promote immunogenic cell death (ICD), releasing tumor-associated antigens and activating dendritic cells (DCs), thereby further supporting their integration with immunotherapy [12, 13]. These mechanistic synergies are supported by clinical trials, including IMbrave150 (atezolizumab + bevacizumab) and KEYNOTE-524 (lenvatinib + pembrolizumab), which have demonstrated improved survival outcomes compared to monotherapy in HCC patients [14, 15]. Nevertheless, a deeper understanding of the cellular interactions driving these combinations remains limited. Given the immunologic, genetic, and epigenetic heterogeneity of HCC, there is a pressing need to dissect how various treatment modalities reshape the TME at the cellular level.

This review synthesizes current knowledge on the mechanisms of combination immunotherapy in HCC, with a focus on interactions among cancer cells, T cells, DCs, and immunosuppressive populations such as regulatory T cells (Tregs), myeloid-derived suppressor cells (MDSCs), and tumor-associated macrophages (TAMs). We further discuss clinical efficacy, current limitations, emerging biomarkers, and technological advances, aiming to deliver the rational design of more effective and personalized combination therapies for HCC.

## Immunotherapy in HCC: a cellular perspective

### The immune landscape of HCC

HCC develops in a chronically inflamed and immunosuppressive microenvironment that impairs antitumor immunity, which contributes to its classification as a poorly immunogenic tumor. Persistent inflammation leads to the accumulation of immunosuppressive cells, fibrotic remodeling, and antigen overstimulation, all of which suppress effective immune surveillance and promote tumor progression. This complex immune landscape—marked by T cell dysfunction, inhibitory cell populations, and physical barriers to immune infiltration—creates a tumor-permissive environment that limits the effectiveness of immunotherapy and underscores the need for combinatorial approaches to overcome immune resistance.

Among the most well-characterized consequences of this immunosuppressive environment is the progressive dysfunction of cytotoxic CD8⁺ T cells, a central obstacle to effective antitumor immunity in HCC. Continuous antigen exposure drives T cells into an exhausted state, marked by reduced cytokine production, impaired proliferation, and diminished cytotoxic activity. This exhaustion is reinforced by the upregulation of multiple immune checkpoints, including programmed cell death protein-1 (PD-1), cytotoxic T-lymphocyte-associated protein 4 (CTLA-4), lymphocyte activation gene-3 (LAG-3) and T cell immunoglobulin domain and mucin domain-3 (TIM-3), all of which suppress effector function and promote immune tolerance within the TME [16, 17]. These exhausted T cells are frequently expressed on tumor-infiltrating lymphocytes in HCC patients, fail to eliminate tumor cells effectively, and often coexist with immunosuppressive signals that further dampen antitumor responses.

In addition to T cell exhaustion, the HCC TME is enriched with immunosuppressive cell populations, such as Tregs, MDSCs, and TAMs, particularly the M2-like phenotype, which further disrupts antitumor immunity. Tregs suppress CD8^+^ T cell activity through multiple mechanisms, including secretion of immunosuppressive cytokines interleukin-10 (IL-10) and transforming growth factor-beta (TGF-β), and IL-35, as well as high expression of CTLA-4, which impairs antigen presentation by DCs [18, 19]. Tregs also consume IL-2 by expressing high levels of CD25, limiting its availability to effector T cells and inhibiting their proliferation. Furthermore, Tregs express the ectonucleotidases CD39 and CD73, which convert extracellular adenosine triphosphate (ATP) into the immunosuppressive adenosine, further dampening T cell responses [20]. Elevated infiltration of CD39⁺ T cells has been associated with reduced OS in patients with HCC [21].

MDSCs inhibit T cell activation and expansion by depleting L-arginine through expression of inducible nitric oxide synthase (iNOS) and arginase 1 (ARG1). They also produce reactive oxygen and nitrogen species (ROS/RNS), which interfere with T cell receptor signaling, leading to impaired T cell activation and proliferation, and depletion of L-arginine can enhance this effect [22]. In addition, MDSCs express immune checkpoint Ligands such as programmed cell death-ligand 1 (PD-L1) and galectin-9, which interact with PD-1 and TIM-3 on T cells, respectively, contributing to T cell exhaustion, and they promote the expansion of Tregs through secretion of IL-10 and TGF-β [9, 23].

TAMs in HCC typically adopt an M2-like phenotype, especially in advanced stages of the disease, and support tumor progression by promoting angiogenesis, tissue remodeling, and immune evasion. Like MDSCs, TAMs secrete IL-10, TGF-β, and VEGF, express PD-L1 and galectin-9, and facilitate the induction of Tregs [9, 24]. Although recent single-cell RNA sequencing (scRNA-seq) studies have identified TAM populations that co-express both M1- and M2-associated gene signatures, the immunosuppressive functions of M2-like TAMs remain dominant and are associated with a poor prognosis [25].

**Fig. 1:**
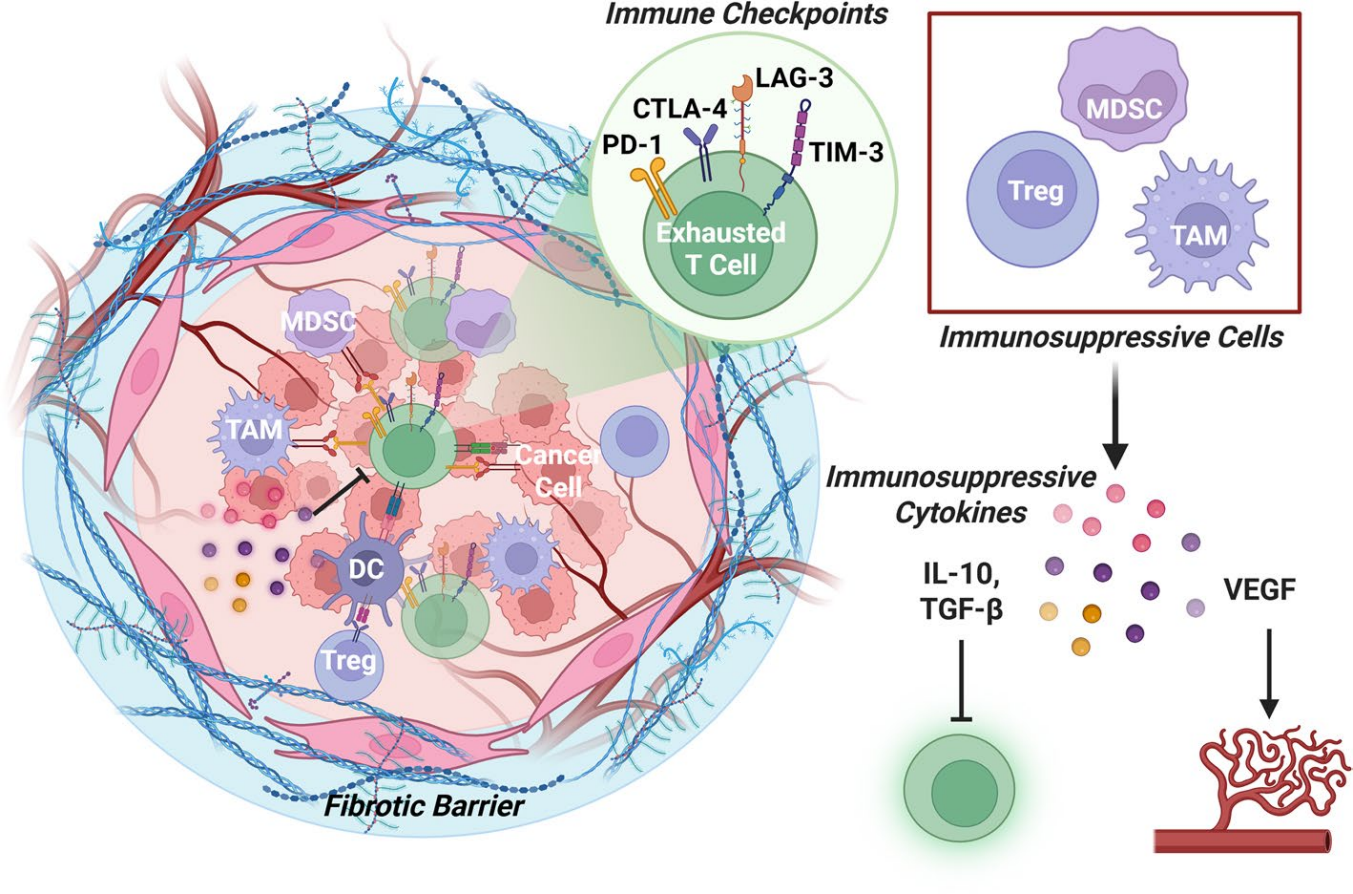
The immunosuppressive TME in HCC. In HCC, cytotoxic CD8⁺ T cells exhibit an exhausted phenotype characterized by upregulation of immune checkpoints including PD-1, CTLA-4, LAG-3, and TIM-3. The TME is populated by immunosuppressive cells, including Tregs, MDSCs, and TAMs, which engage immune checkpoints and secrete IL-10, TGF-β, and VEGF to suppress T cell function and promote tumor progression. A dense fibrotic stroma further impairs immune infiltration, contributing to an immune-excluded phenotype

Beyond cellular immunosuppression, fibrotic remodeling of the TME creates a physical barrier that limits immune cell infiltration. The dense extracellular matrix restricts the access of cytotoxic CD8⁺ T cells to the tumor core and contributes to an immune-excluded phenotype, in which immune cells are trapped in the peritumoral stroma without engaging tumor cells directly[26]. This spatial dislocation further impairs immune surveillance and blunts the effectiveness of immune checkpoint blockade (Fig. 1).

PD-L1 is frequently overexpressed in HCC, allowing tumor and immune cells to engage with PD-1 on exhausted T cells and suppress their function [27, 28]. Although blockade of the PD-1/PD-L1 axis can partially restore T cell activity, many tumors retain resistance through compensatory mechanisms, such as the upregulation of alternative checkpoints (e.g., LAG-3, TIM-3) or the activation of immunosuppressive metabolic pathways [16]. Additionally, substantial heterogeneity exists across HCC tumors in terms of immune cell infiltration and TME composition. This heterogeneity—ranging from immune-inflamed to immune-excluded or immune-desert phenotypes—contributes to the variability in immunotherapy responsiveness and highlights the need for combination strategies that can enhance immune activation and improve clinical outcomes [29].

### Mechanisms of ICIs in HCC

ICIs work by reactivating cytotoxic T lymphocytes and overcoming the immunosuppressive barriers that characterize the HCC TME. These barriers, including inhibitory cytokines, checkpoint ligand expression, and restricted T-cell infiltration, contribute to immune evasion. By targeting the PD-1/PD-L1 and CTLA-4 pathways, ICIs help restore antitumor immunity and promote more effective immune-mediated tumor elimination.

PD-1 is a key inhibitory receptor expressed on exhausted T cells. In HCC, its ligand, PD-L1, is frequently upregulated on tumor cells and immunosuppressive cells, such as MDSCs and TAMs. Engagement of PD-1 by PD-L1 inhibits T cell proliferation, cytokine production, and cytolytic function. ICIs targeting this axis—such as nivolumab and pembrolizumab—block the PD-1/PD-L1 interaction, thereby reinvigorating T cell activity and restoring effector function within the TME [30]. High tumor PD-L1 expression has been associated with improved responses to PD-1 blockade; however, clinical benefit is also observed in some patients with low PD-L1 levels [31, 32].

CTLA-4 is another inhibitory receptor, primarily expressed on Tregs and activated conventional T cells. It competes with the costimulatory receptor CD28 for binding to B7 molecules (CD80/CD86) on antigen-presenting cells (APCs). By outcompeting CD28, CTLA-4 dampens the priming and activation of naïve T cells. Inhibitors such as ipilimumab and tremelimumab block CTLA-4 signaling, thereby enhancing T-cell priming and reducing Treg-mediated suppression [33, 34]. This mechanism is especially relevant in early phases of T cell activation within lymphoid tissues and may synergize with PD-1 blockade in tumors.

**Fig. 2:**
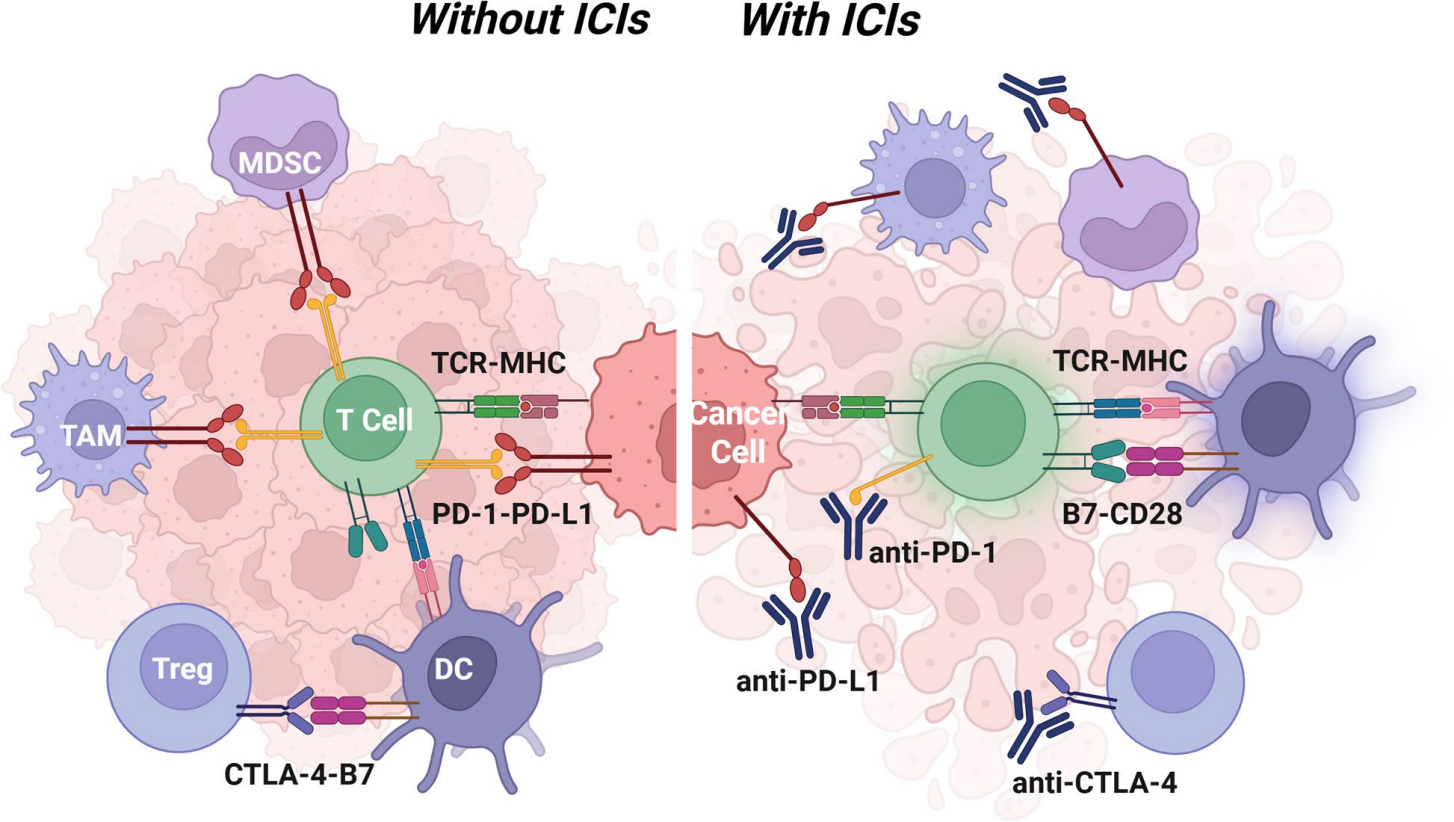
Mechanisms of action of ICIs in HCC. ICIs restore antitumor immunity by blocking key inhibitory pathways that suppress T cell function. PD-1, expressed on exhausted T cells, binds PD-L1 on tumor and immune cells such as MDSCs and TAMs, leading to T cell inhibition. Anti-PD-1/PD-L1 therapies (e.g., nivolumab, pembrolizumab) disrupt this interaction, thereby reinvigorating T cell cytotoxicity. CTLA-4, primarily found on Tregs and activated T cells, competes with CD28 for binding to B7 molecules on antigen-presenting cells, thereby limiting T cell priming. CTLA-4 inhibitors (e.g., ipilimumab, tremelimumab) block this suppression and enhance T cell activation in lymphoid tissues. Together, these ICIs enhance effector T cell activity and shift the TME toward an immune-responsive state

Beyond direct effects on T cells depicted in Fig. 2, ICIs have also been shown to reshape the broader immune landscape in HCC. Preclinical and clinical studies suggest that PD-1 and CTLA-4 blockade can reduce the frequency of Tregs and MDSCs in the TME while increasing infiltration of effector CD8⁺ T cells [35]. However, ORR with ICI monotherapy remains modest (approximately 15%–20%) due to adaptive resistance mechanisms, such as compensatory upregulation of LAG-3 or TIM-3, increased TGF-β signaling, and continued recruitment of immunosuppressive myeloid cells [4, 5, 36].

Given these limitations, combination therapies are being actively explored. These include pairing ICIs with TKIs to normalize tumor vasculature and reduce VEGF-mediated suppression, combining ICIs with chemotherapy to increase tumor antigen release and DC activation, or using dual ICI regimens to target multiple immune checkpoints simultaneously. Understanding the specific immunologic barriers targeted by each combination strategy is crucial for enhancing patient outcomes and tailoring personalized treatment regimens.

## Combination therapy strategies and their cellular mechanisms

### ICIs + TKIs

#### Immune and vascular barriers to ICI efficacy in HCC

The rationale for combining TKIs with ICIs in HCC is grounded in their complementary mechanisms of action within the TME. HCC is a highly vascularized malignancy characterized by aberrant angiogenesis, contributing to immunosuppressive TME [10]. These features not only support tumor progression but also hinder the effectiveness of immunotherapy. TKIs, initially developed for their anti-angiogenic properties, have since been shown to possess immune-modulatory effects that can enhance the efficacy of ICIs.

A Major driver of immunosuppression in HCC is hypoxia, which arises from highly active Angiogenesis. Hypoxic conditions stabilize hypoxia-inducible factor 1-alpha (HIF-1α), leading to upregulation of VEGF. VEGF promotes disorganized, aberrant vasculature that impedes immune cell infiltration, while also recruiting immunosuppressive cell populations, such as MDSCs and TAMs [9]. These myeloid populations further inhibit cytotoxic T cell activity and contribute to resistance against ICIs.

TKIs, such as lenvatinib and cabozantinib, inhibit VEGF and its receptor signaling, thereby reducing pathological angiogenesis and partially normalizing tumor vasculature and immunosuppression within the TME [2]. This vascular normalization alleviates hypoxia, facilitates T cell infiltration, and shifts the immune balance toward a more inflamed phenotype [37, 38]. In addition, TKIs suppress the production of immunosuppressive cytokines, including IL-10 and TGF-β, which are key mediators of immune suppression and promote fibrosis in the HCC microenvironment [39]. Beyond their vascular effects, TKIs also modulate antigen presentation. Preclinical and clinical studies have demonstrated that VEGF blockade enhances the maturation of DCs and improves antigen presentation, thereby making tumor cells more visible to the adaptive immune system [40, 41]. These changes further support T cell priming and effector function, amplifying the therapeutic benefit of ICIs. Importantly, certain TKIs target additional oncogenic and immunosuppressive pathways beyond VEGF. For example, cabozantinib inhibits the mesenchymal-epithelial transition factor (MET) and anexelekto (AXL), both of which are implicated in immune evasion, epithelial–mesenchymal transition, and tumor invasiveness in HCC [42]. These additional targets contribute to broader immune reprogramming and may sensitize tumors to immunotherapy.

While most evidence focuses on VEGF signaling, there is emerging data suggesting that epidermal growth factor receptor (EGFR) inhibitors (EGFR-TKIs) may also enhance ICI efficacy. In non-small-cell lung cancer (NSCLC), for example, EGFR-TKIs have been shown to downregulate PD-L1 expression, potentially sensitizing tumors to PD-1/PD-L1 blockade. Although these findings have not been fully validated in HCC, they highlight the potential of pathway-specific TKIs to modulate immune checkpoints and influence the responsiveness to ICI. Taken together, the integration of TKIs with ICIs is supported by robust mechanistic evidence. By simultaneously targeting tumor angiogenesis, cytokine-mediated immunosuppression, antigen presentation deficits, and oncogenic signaling, TKIs create a more permissive environment for the immune system. This synergy provides a strong foundation for combination strategies and opens the door to personalized therapeutic approaches in HCC.

#### Mechanisms of synergy between TKIs and ICIs

The synergy between TKIs and ICIs in HCC arises from a cascade of interconnected changes within the TME, where TKIs modulate vascular, cellular, and antigen-presenting conditions that directly enhance the immune-activating potential of ICIs. TKIs, particularly those targeting VEGF signaling, remodel the TME by correcting vascular abnormalities, alleviating hypoxia, and reducing immunosuppressive cell populations, thereby enhancing the functional impact of ICIs. These effects span multiple pathways, including improved DC function, reduced regulatory and myeloid suppressor cell activity, vascular normalization, and enhanced antigen availability, each contributing to a more immunogenic TME.

One critical point of convergence is the hypoxia-VEGF axis. Under hypoxia, HCC tumors upregulate VEGF through the HIF-1α pathway, which promotes chaotic angiogenesis and hinders immune cell infiltration [43]. VEGF blockade by TKIs restores vascular integrity, alleviates hypoxia, and allows cytotoxic T cells to access tumor tissue. Moreover, this reoxygenation effect reprograms TAMs, shifting them from an immunosuppressive M2-like phenotype toward a pro-inflammatory M1 state that supports antitumor responses of ICIs [44]. TKIs also suppress the accumulation and function of MDSCs, which otherwise inhibit T cell activity and secrete immunosuppressive factors. Cabozantinib and lenvatinib have both been shown to reduce MDSC levels, thereby removing a major barrier to immune activation. Similarly, TKIs reduce Treg infiltration within the tumor, thereby alleviating one of the primary sources of local immunosuppression and enhancing the responsiveness of ICIs [45].

**Fig. 3:**
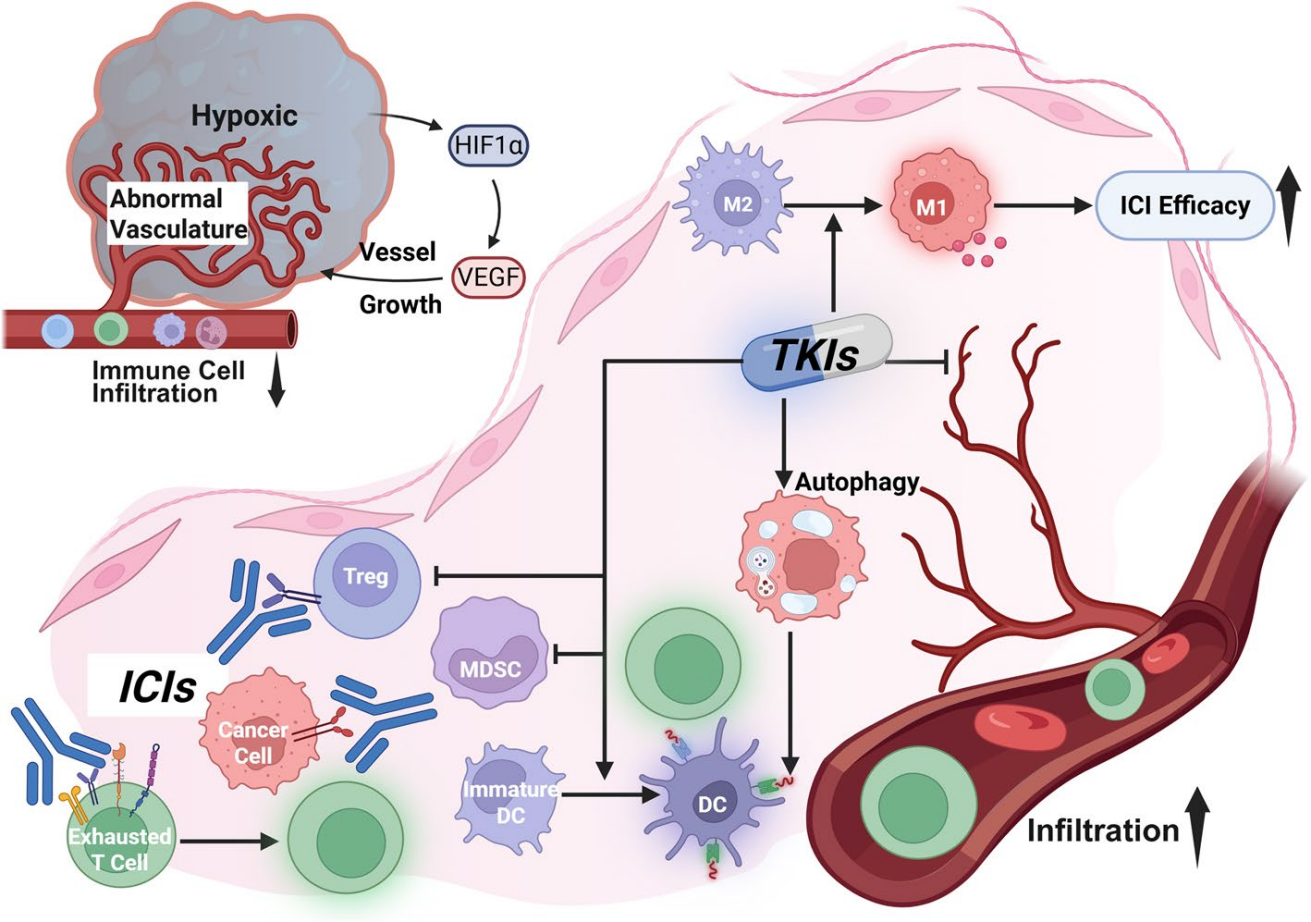
Synergistic mechanisms of ICI–TKI therapy. Hypoxia-induced VEGF overexpression leads to the development of abnormal vasculature, immune exclusion, and the recruitment of immunosuppressive cells, including MDSCs, Tregs, and M2-like TAMs. TKIs normalize tumor vasculature, alleviate hypoxia, and reduce these immunosuppressive populations, improving immune infiltration. They also enhance DC maturation and promote antigen availability through autophagy induction. When combined with ICIs, these changes reinvigorate CD8⁺ T cell responses, leading to more effective tumor control

Antigen presentation is another point of mechanistic synergy. VEGF inhibition promotes the maturation of DCs, thereby enhancing their ability to present tumor antigens and prime effector T cells [40, 41]. Some TKIs extend this effect by inducing autophagy in tumor cells. For example, EGFR-targeting TKIs enhance the release of extracellular antigens, which DCs can then process and present, thereby further amplifying CD8^+^ T cell responses [46, 47]. Preclinical data support these interlinked effects. In murine models of HCC, the combination of VEGF inhibition and PD-1 blockade leads to increased intratumoral CD8^+^ T cell infiltration and more pronounced tumor regression than either therapy alone [48–50]. This supports the idea that TKIs do not merely improve the delivery of ICIs but actively transform the immune landscape to favor ICI activity (Fig. 3).

However, the benefits of ICI-TKI therapy are not uniformly observed across all patients. Variability in tumor immune phenotype and intrinsic biological features may influence treatment response. Biomarkers such as VEGF expression levels [51], high tumor mutational burden (TMB) [52], and tumor-infiltrating lymphocyte (TIL) density [53] are being investigated as tools to predict which patients are most likely to benefit from combination strategies. Personalized selection based on these factors may further enhance clinical outcomes.

#### Key ICI-TKI combinations in HCC

Multiple clinical trials have established the efficacy of ICI–TKI combinations in hepatocellular carcinoma, with several regimens now approved as first-line therapies (Table 2). These combinations leverage the immune-modulatory effects of TKIs to enhance the efficacy of checkpoint blockade and have demonstrated consistent survival benefits across diverse patient populations.

**Table 2:** Key clinical trials of ICI-TKI combinations in HCC

ClinicalTrials.gov	Trial Name	Combination Therapy	Phase	Primary Endpoint	Median OS [Months]	Median PFS [Months]	ORR☆	mDOR☆ [Months]	TRAE Grade ≥ 3	Enrolled Cohort (n)	The Most Common Grade 3–4 Drug-related Side Effects	Indication	References
NCT03434379	IMbrave150	atezolizumab + bevacizumab vs. sorafenib	Phase 3	OS, PFS	NE vs. 13.2	6.8 vs. 4.3	27.3% vs.11.9%	> 6: 87.6% vs. 59.1%	61.1% vs. 60.9%	501	Hypertension: 15.2% vs. 12.2% AST increase: 7.0% vs. 5.1% ALT increase: 3.6% vs. 1.3%	First-line therapy	[14]
				Updated results	19.2 vs. 13.4	6.9 vs. 4.3	30% vs. 11%	18.1 vs. 14.9	45% vs. 47%				[54]
NCT03794440	ORIENT-32	sintilimab + bevacizumab biosimilar (IBI305) vs. sorafenib	Phase 2/3	OS, PFS	NR vs. 10.4	4.6 vs. 2.8	21% vs. 4%	NE vs. 9.8	35% vs. 37%	571	Hypertension: 15% vs. 6% Decreased platelet count: 8% vs. 3% Proteinuria: 5% vs. 2% Increased γ-glutamyltransferase: 5% vs. 2%	First-line therapy	[55]
				Updated results			21.0% vs. 4.7%	20.3					[56]
NCT03764293	CARES-310	camrelizumab + rivoceranib vs. sorafenib	Phase 3	OS, PFS	22.1 vs. 15.2	5.6 vs. 3.7	25% vs. 6%	14.8 vs. 9.2	81% vs. 52%	543	Hypertension: 38% vs. 15% AST increase: 16& vs. 15% ALT increase: 13% vs. 3% Palmar-plantar erythrodysaesthesia syndrome: 12% vs. 15%	First-line therapy	[57]
NCT03713593	LEAP-002	lenvatinib + pembrolizumab vs. lenvatinib	Phase 3	OS, PFS	21.2 vs. 19.0	8.2 vs.8.1	26.1% vs. 17.5%	16.6 vs. 10.4	63% vs. 57%	794	Hypertension: 17% vs. 17% AST increase: 7% vs. 4% Diarrhoea: 6% vs.4%	First-line therapy	[59]
NCT01658878	CheckMate 040, cohort 6	cabozantinib + nivolumab	Phase 2/3	Safety, Tolerability, ORR, DOR	20.2	5.1	17%	8.3	50%	36	Diarrhea: 11% Hypertension: 11% AST increase: 8%	First- or second-line therapy	[60]
**Generic Name**	**Target**	**Brand Name**	**Company**										
Atezolizumab	PD-L1	Tecentriq	Genentech/Roche										
Bevacizumab	VEGF	Avastin	Genentech/Roche										
Sintilimab	PD-1	Tyvyt	Innovent Biologics and Eli Lilly										
IBI305	VEGF (Bevacizumab biosimilar)	Byvasda	Innovent Biologics										
Camrelizumab	PD-1	AiRuiKa	Jiangsu Hengrui Medicine										
Rivoceranib	VEGFR-2	Apatinib	Jiangsu Hengrui Medicine (China) Elevar Therapeutics (global)										
Lenvatinib	VEGFR1-3, FGFR1-4, PDGFRα, RET, KIT	Lenvima	Eisai										
Pembrolizumab	PD-1	Keytruda	Merck										
Cabozantinib	VEGFR-2, MET, AXL	Cometriq (thyroid cancer); Cabometyx (renal cell carcinoma)	Exelixis (U.S.); Takeda and Ipsen (ex-U.S.)										
Nivolumab	PD-1	Opdivo	Bristol Myers Squibb (BMS) and Ono Pharmaceutical										

*OS* overall survival, *PFS* progression-free survival, *ORR* objective response rate, *DOR* duration of response, *NE* not estimable, *NR* not reached, *TRAE* treatment-related adverse events

☆ According to the Response Evaluation Criteria in Solid Tumors, version 1.1 (RECIST 1.1)

The IMbrave150 trial (NCT03434379) marked a turning point in first-line HCC therapy. Atezolizumab (anti–PD-L1) combined with bevacizumab (anti–VEGF-A) improved OS (19.2 vs. 13.4 months) and progression-free survival (PFS) (6.9 vs. 4.3 months) compared to sorafenib, with an ORR of 30% (RECIST v1.1) [14, 54]. Mechanistically, bevacizumab reduces VEGF-mediated immunosuppression and abnormal vasculature, while atezolizumab reactivates exhausted T cells. Together, these agents promote vascular normalization, enhance immune infiltration, and re-establish antitumor immunity, forming the rationale for dual inhibition of angiogenesis and immune checkpoints.

Building on this VEGF-PD-1/PD-L1 targeting strategy, the ORIENT-32 trial (NCT03794440) evaluated sintilimab (anti–PD-1) with IBI305, a bevacizumab biosimilar. While similar in concept to IMbrave150, this combination targets PD-1 directly on T cells, potentially broadening immune reactivation. The trial showed a comparable OS benefit (not estimable vs. 10.4 months) and an ORR of 21% (RECIST v1.1) [55, 56], further validating VEGF blockade as a platform for ICIs efficacy across PD-1/PD-L1 pathways. Based on the ORIENT-32 trial, the combination of Tyvyt® (sintilimab) and Byvasda® (bevacizumab) has been approved by the National Medical Products Administration (NMPA) for the first-line treatment of unresectable or metastatic HCC in China.

Expanding the landscape to include a VEGFR inhibitor, the CARES-310 trial (NCT03764293) combined camrelizumab (anti–PD-1) with rivoceranib (apatinib), a selective VEGFR2 inhibitor. VEGFR2 plays a central role in angiogenesis, and its inhibition disrupts the recruitment of suppressive myeloid cells, thereby supporting DC activation. Camrelizumab reactivates CD8⁺ T cells in parallel. This combination Yielded an OS of 22.1 months and an ORR of 25% (RECIST v1.1) [57], leading to NMPA approval and inclusion of first-line treatment recommendations in the ESMO Clinical Practice Guideline (2025) [58]. Compared to monoclonal VEGF antibodies, small-molecule TKIs, such as rivoceranib, may offer broader vascular remodeling and intracellular signaling disruption.

Further broadening the mechanistic scope, KEYNOTE-524 (NCT03006926) explored the combination of pembrolizumab (anti–PD-1) with Lenvatinib, a multikinase inhibitor targeting VEGFR1-3, FGFR1-4, RET, KIT, and PDGFRα. This combination acts on both angiogenesis and Tumor-intrinsic growth pathways, potentially sensitizing immune-desert Tumors through modulation of fibroblast signaling and stromal architecture. The study reported an ORR of 46% (mRECIST per IIR) or 36% (RECIST v1.1), the highest among early-phase ICI–TKI trials [15]. However, in the Phase 3 LEAP-002 trial (NCT03713593), the combination narrowly missed statistical significance compared to lenvatinib alone, likely due to unexpectedly strong outcomes in the control arm [59]. These findings underscore the need for biomarker-guided patient stratification.

Targeting a different resistance axis, CheckMate 040 cohort 6 (NCT01658878) combined nivolumab (anti–PD-1) with cabozantinib, which inhibits not only VEGFR but also MET, AXL, and RET—key drivers of epithelial–mesenchymal transition, immune exclusion, and stromal remodeling. This multi-targeted approach may benefit patients with immune-excluded or mesenchymal Tumors that are poorly responsive to conventional ICI therapy. The trial reported a median OS of 20.2 months and an ORR of 17% (RECIST v1.1) [60]. The inclusion of MET/AXL inhibition suggests added value in reprogramming immune-resistant microenvironments.

### ICIs + chemotherapy

#### Mechanistic rationale: how chemotherapy primes the immune response

While traditionally known for their cytotoxic effects, certain chemotherapy agents can also initiate ICD, transforming tumors into sources of antigens and immune stimulators. ICD is characterized by the release of danger-associated molecular patterns (DAMPs), such as calreticulin, ATP, and high mobility group box 1 protein (HMGB1), which promote the activation of DCs and antigen presentation, ultimately supporting more robust T cell responses [61, 62]. For example, oxaliplatin, a platinum-based agent, has demonstrated the ability to induce ICD and enhance DC-mediated cross-priming of antigen-specific CD8^+^ T cells in HCC models [63].

Beyond antigen release, chemotherapy can modulate the TME by reducing the populations of immunosuppressive cells. Agents such as 5-FU [64] and doxorubicin [65] have been reported to deplete MDSCs, which normally suppress cytotoxic T cell activity and sustain an immunosuppressive niche. Paradoxically, a recent study indicated that 5-FU may also promote the recruitment of MDSCs, ultimately diminishing the efficacy of PD-L1 blockade in HCC [66]. This paradox highlights the unique immunobiology of the liver and underscores the need for cancer-specific evaluation of immunomodulatory effects.

Similarly, chemotherapy agents such as cyclophosphamide (CTX) [67] and gemcitabine [68] can  selectively deplete Tregs, relieving inhibitory pressure on T cells and enhancing the potential for ICIs to function effectively. Murine models and clinical trials support the notion that chemotherapy augments PD-1 blockade efficacy, resulting in more robust tumor regression than either therapy alone [48, 69, 70]. Nonetheless, not all chemotherapies confer immune benefit. Certain agents, including paclitaxel (PTX) [71] and high-dose CTX [72], may act in an immunosuppressive rather than immunostimulatory fashion, reinforcing the need for careful selection and dosing to avoid undermining ICI activity.

#### Immune synergies and TME remodeling

Chemotherapy can reshape the TME in ways that amplify the efficacy of ICIs. By inducing necrosis and apoptosis in tumor cells, chemotherapy promotes the release of tumor antigens, thereby increasing the visibility of cancer cells to the immune system [73]. This effect is compounded by reductions in MDSCs and Tregs, which shift the TME from an immunosuppressive to an immunostimulatory state [74]. In addition to cellular changes, chemotherapy alters the cytokine milieu. CTX, for example, has been shown to suppress TGF-β and IL-10 while enhancing the secretion of pro-inflammatory cytokines, such as interferon-gamma (IFN-γ) and IL-12 [75]. These changes help generate a TME that is more conducive to immune activation. ICD-inducing agents, including CTX, oxaliplatin, and anthracyclines, promote DCs'maturation and facilitate cross-presentation of tumor antigens, strengthening adaptive immunity [76].

**Fig. 4:**
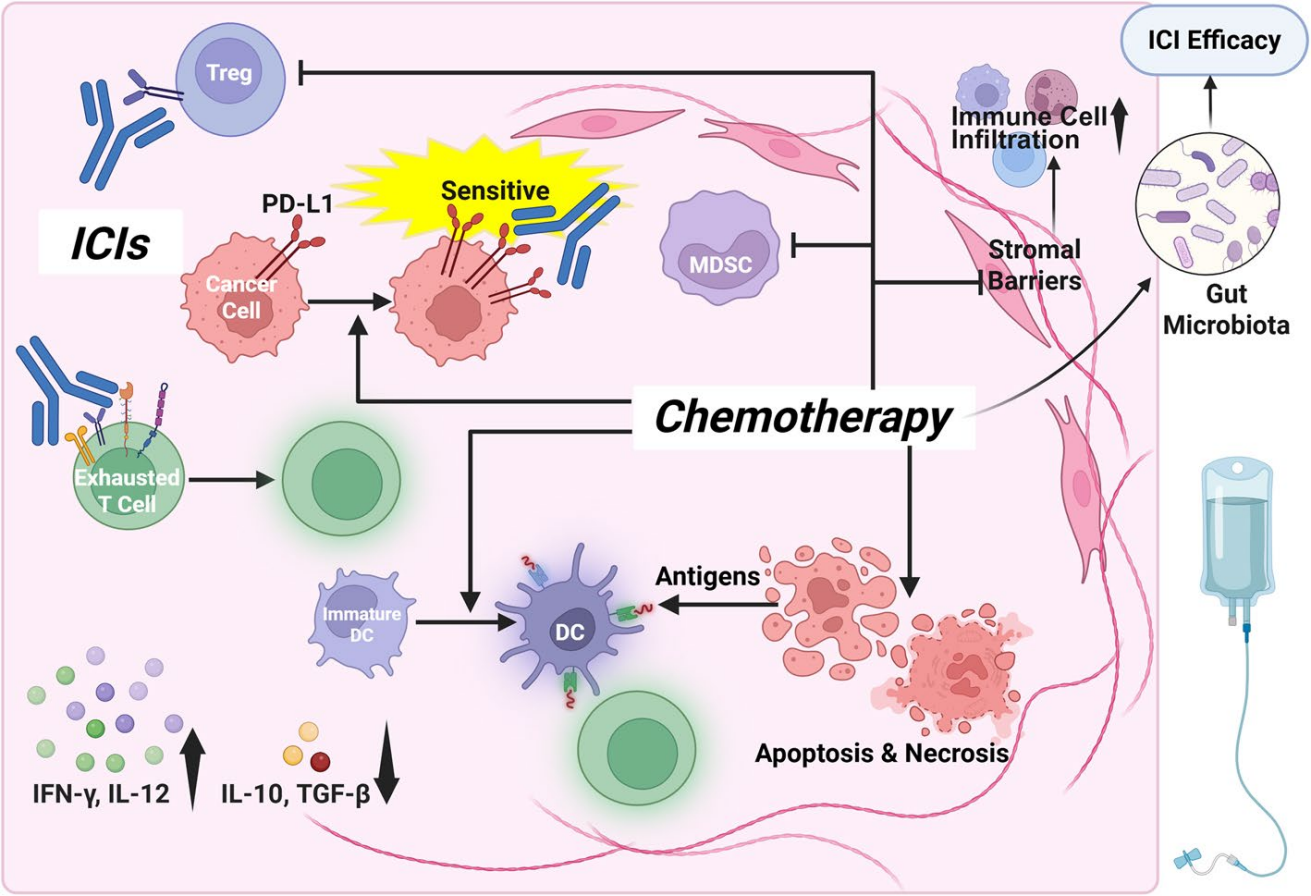
ICD and ICI-chemotherapy synergy. Chemotherapy induces tumor cell death and releases antigens, aiding DC activation and T cell priming. It depletes suppressive populations, such as Tregs and MDSCs, remodels stromal barriers to facilitate immune infiltration, and alters cytokine balance to favor immune stimulation. Some agents also increase PD-L1 expression or shift gut microbiota composition, further enhancing ICI sensitivity

Chemotherapy can also remodel the tumor architecture. By reducing desmoplasia and stromal density, certain agents, such as losartan and pentoxifylline, enhance immune cell infiltration [77–79]. Interestingly, cisplatin has been observed to increase PD-L1 expression on tumor cells, potentially enhancing their susceptibility to PD-1/PD-L1 blockade [80]. Emerging evidence also suggests that chemotherapies, such as 5-FU and CTX, may influence gut microbiota composition, which in turn modulates systemic immune responses and immunotherapy sensitivity [81–84]. Notably, the sequence of chemotherapy relative to ICI administration, whether administered before, during, or after, may influence the degree of immune synergy achieved [85]. Given the mechanisms demonstrated in Fig. 4, several chemotherapy agents have been explored in combination with ICIs in clinical trials for HCC.

#### Clinical evidence: trials of ICI–chemotherapy combinations in HCC

Multiple clinical trials have investigated the combination of chemotherapy with ICIs to enhance response rates in HCC. Clinical trials are exploring various chemotherapy agents in combination with ICIs to improve response rates in HCC patients. Platinum-based oxaliplatin is a leading candidate due to its strong ICD-inducing properties. It enhances DCs activation, enhances antigen presentation, and increases immune cell infiltration, thereby complementing PD-1 blockade [63]. Hepatic artery infusion chemotherapy (HAIC) combined with anti-PD-1 therapy has shown better clinical outcomes than HAIC alone [69]. A phase II study (NCT03092895) assessing oxaliplatin-based chemotherapy plus camrelizumab reported a 26.5% ORR with manageable toxicity; grade ≥ 3 immune-related adverse events (irAEs) were observed in only 5.9% of patients [86].

Capecitabine, a 5-FU prodrug, also holds promise. It modulates immune cell populations and promotes a pro-inflammatory TME, increasing tumor susceptibility to ICIs. In a phase II clinical study (NCT04411706), a triplet regimen of capecitabine, sintilimab, and rivoceranib demonstrated a 50% ORR, with 28.3% of patients experiencing grade ≥ 3 treatment-related adverse events (TRAEs) [87]. However, not all chemotherapeutics deliver similar immunologic benefits. The variability in immune effects among agents—and even among different doses of the same agent—necessitates careful selection based on immune-modulatory profiles. Dosing, sequencing, and toxicity management remain active areas of research to fully harness the therapeutic potential of ICI-chemotherapy combinations in HCC.

### Dual ICIs (PD-1/PD-L1 + CTLA-4)

#### Mechanistic rationale: complementary roles of PD-1 and CTLA-4 blockade

The rationale for dual ICI therapy, which combines PD-1/PD-L1 and CTLA-4 inhibitors, lies in their distinct yet complementary roles in regulating T cells. PD-1/PD-L1 primarily functions in the late effector phase, limiting T cell activity within the TME, whereas CTLA-4 regulates earlier stages of T cell activation during priming of the lymphoid tissues. By simultaneously targeting both checkpoints, dual ICI therapy enhances both the initiation and execution of antitumor immunity.

CTLA-4 blockade (e.g., ipilimumab, tremelimumab) enhances antigen presentation and T cell priming by disrupting CTLA-4-mediated suppression of DCs, allowing greater CD28-B7 co-stimulation. In contrast, PD-1/PD-L1 blockade (e.g., nivolumab, durvalumab) restores the function of exhausted T cells within the TME, thereby enhancing cytotoxic responses against tumor cells [88, 89]. Preclinical studies support this dual mechanism. In murine models of HCC, simultaneous blockade of PD-1 and CTLA-4 led to significantly enhanced tumor regression compared to either therapy alone [90]. These findings are supported clinically by the CheckMate 9DW trial, where nivolumab plus ipilimumab demonstrated improved response rates over monotherapy [91].

Dual checkpoint inhibition may be particularly effective in HCC tumors with an"immune-inflamed"phenotype, characterized by existing T cell infiltration and activation, whereas immune-desert tumors may require additional priming strategies. Although dual blockade is associated with higher rates of irAEs, optimized dosing regimens (e.g., a single CTLA-4 priming dose) are being developed to minimize toxicity without compromising efficacy [92].

#### Immune synergies and functional outcomes of dual blockade

Dual immune checkpoint blockade enhances antitumor immunity by targeting complementary stages of T cell regulation. CTLA-4 inhibition promotes T cell priming in secondary lymphoid organs by blocking CTLA-4's suppression of CD28-mediated co-stimulation, thereby increasing the proliferation and activation of naïve T cells. Concurrently, PD-1/PD-L1 blockade reverses T cell exhaustion within the TME, restoring cytotoxic activity, cytokine production, including IFN-γ and tumor necrosis factor-alpha (TNF-α), and tumor cell killing [93, 94]. This dual mechanism supports both the initiation and effector phases of T cell responses, contributing to more durable tumor control.

Importantly, dual checkpoint inhibition extends beyond CD8⁺ T cells. Natural killer (NK) cells expressing PD-1, especially activated subsets marked by Sca-1⁺ and CD69⁺, exhibit enhanced cytotoxicity following PD-1 blockade, further amplifying the antitumor immune treatment response [95]. Additionally, CTLA-4 inhibitors have been shown to reduce intratumoral Treg populations in murine models via Fc-mediated depletion [96]. However, this effect is less consistent in human tumors, prompting the development of optimized antibody formats to enhance Treg-targeting capacity [97].

**Fig. 5:**
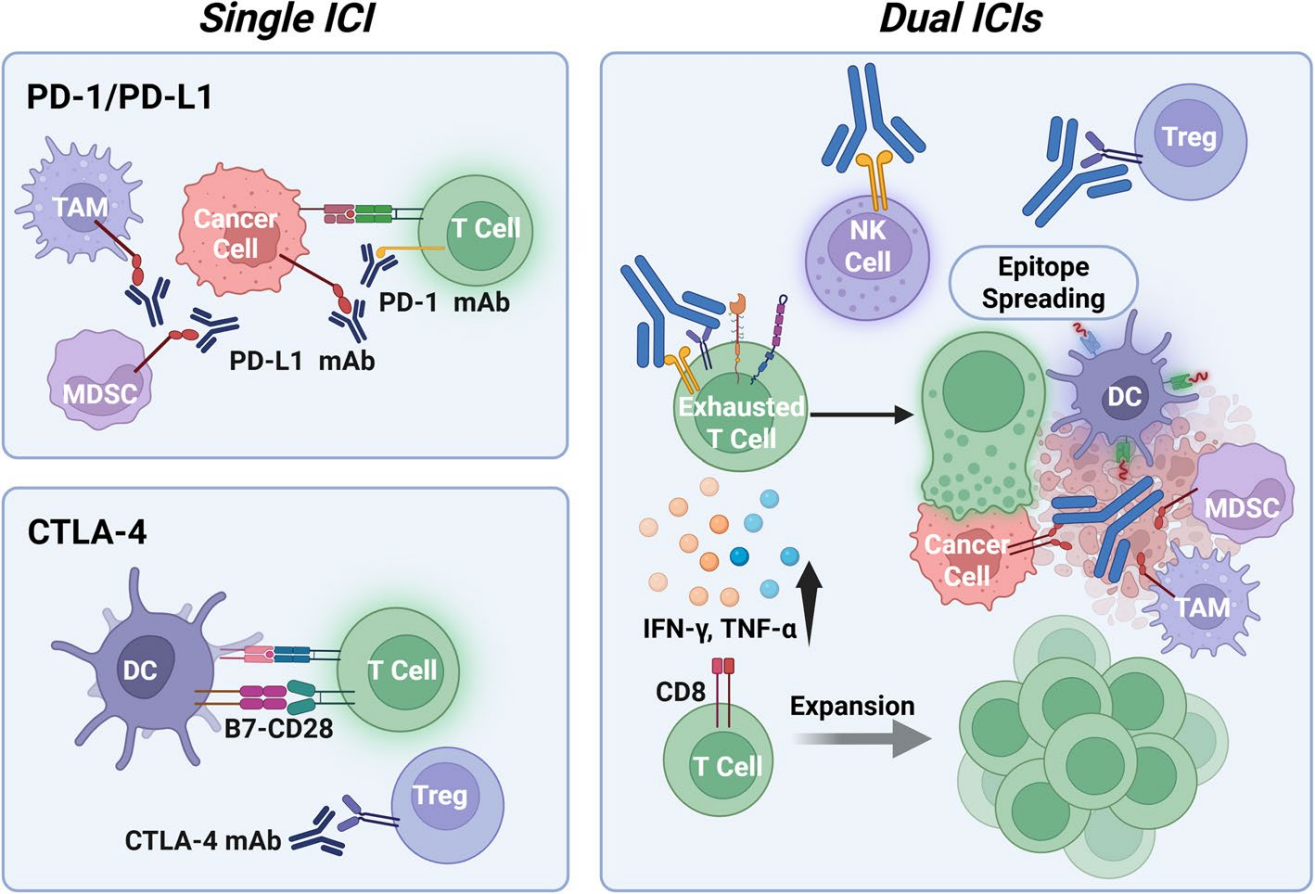
Mechanistic synergies of dual immune checkpoint blockade in HCC. Dual inhibition of PD-1 and CTLA-4 enhances antitumor immunity through complementary mechanisms. CTLA-4 blockade promotes early T cell priming in lymphoid tissues by enhancing CD28 co-stimulation, while PD-1 blockade reinvigorates exhausted effector T cells within the tumor. This combination enhances cytokine production (e.g., IFN-γ, TNF-α), expands the function of cytotoxic T and NK cells, and facilitates epitope spreading. Additionally, dual blockade supports the formation of long-lived memory CD8⁺ T cells, contributing to durable tumor control

Another synergistic effect of dual blockade is the phenomenon of epitope spreading, in which the immune response expands beyond initial antigens to target additional neoepitopes. This reduces the risk of immune escape and promotes more comprehensive tumor clearance [98]. Moreover, dual checkpoint inhibition fosters the clonal expansion of effector CD8⁺ T cells and supports the generation of long-term memory subsets. In particular, CTLA-4 inhibition has been associated with an increase in T cell factor-1 (TCF-1)⁺ memory progenitor CD8⁺ T cells, which are crucial for sustained anti-tumor immune surveillance and preventing relapse [99–101] (Fig. 5).

The efficacy of dual ICI therapy appears to be modulated by tumor-intrinsic factors. HCC tumors with high TMB or pre-existing T cell infiltration—hallmarks of the “immune-inflamed” phenotype—tend to respond more favorably to dual checkpoint blockade [102]. However, heightened immune activation also increases the risk of irAEs, including colitis, hepatitis, and endocrinopathies, which pose challenges for broader clinical use [103]. To mitigate toxicity while preserving efficacy, modified dosing regimens—such as intermittent or reduced CTLA-4 inhibition—are under investigation [92]. In parallel, bispecific antibodies have been engineered to co-target PD-1 and CTLA-4 in a structurally optimized and tumor-selective manner. This kind of agent aims to replicate the therapeutic benefits of dual blockade while minimizing off-target immune activation. Ultimately, the advancement of dual ICI therapy in HCC will hinge on biomarker-driven approaches that enable precise patient stratification. Tailoring treatment based on TME characteristics, immune phenotypes, and molecular markers will be critical to maximizing clinical benefit while limiting toxicity in this high-risk population.

#### Clinical evidence: dual ICI strategies in HCC trials

The clinical development of dual ICIs in HCC reflects an evolving strategy to balance efficacy with safety. Across several key trials, co-inhibition of PD-1/PD-L1 and CTLA-4 has consistently demonstrated superior antitumor activity compared to monotherapies; however, challenges related to toxicity and patient selection remain ongoing obstacles to its optimization.

The CheckMate 9DW trial (NCT04039607), a phase Ⅲ clinical trial, evaluated nivolumab (anti–PD-1) in combination with ipilimumab (anti–CTLA-4) in patients with advanced, unresectable HCC. The dual ICI regimen Yielded a median OS of 23.7 months and an ORR of 36%, substantially exceeding the 20.6 months median OS and 13% ORR achieved with lenvatinib or sorafenib [91]. These findings support the clinical relevance of targeting both early and late phases of T cell regulation to enhance antitumor immunity. However, the combination was also associated with a higher incidence of irAEs, including diarrhea and colitis, raising concerns about the tolerability of sustained CTLA-4 inhibition and the need for optimized dosing strategies to improve tolerability [104].

To address these toxicity concerns, the phase 3 HIMALAYA trial (NCT03298451) introduced a modified dosing strategy known as the STRIDE regimen (Single Tremelimumab Regular Interval Durvalumab). This approach involved a single high-dose priming administration of tremelimumab (anti-CTLA-4) followed by regular maintenance with durvalumab (anti-PD-L1). The STRIDE regimen demonstrated improved median OS of 16.4 months vs. 13.8 months with sorafenib or 16.6 months with durvalumab, with an ORR of 20.1% compared to 5.1% or 17.0%. Importantly, this regimen exhibited a more favorable safety profile, leading to its approval by the U.S. FDA for first-line treatment of unresectable HCC [105]. These results underscore the significance of dosing schedules in modulating the therapeutic window of dual ICI therapy, suggesting that transient CTLA-4 blockade may be sufficient to achieve clinical benefit in select patient populations.

Beyond conventional mAb regimens, bispecific antibodies have emerged as a promising next-generation strategy to reduce systemic toxicity while maintaining dual-target engagement. Agents such as QL1706 [106], MEDI5752 [107], and cadonilimab [108] are designed to simultaneously target PD-1 and CTLA-4 in a tumor-selective manner or optimized molecular formats, potentially reducing systemic toxicity without compromising efficacy. Early-phase data suggest that these agents may enhance T cell activation while minimizing irAEs, offering a mechanistically distinct and potentially more tolerable approach to dual immune modulation. Yet, larger trials are needed to confirm their clinical benefit in HCC.

The therapeutic scope of dual ICI strategies is also expanding to include non-conventional immune checkpoint targets. For instance, relatlimab, an anti-LAG-3 antibody, has demonstrated clinical benefit in combination with nivolumab in melanoma and is currently under investigation for HCC [109]. These efforts reflect a broader shift toward multi-target immune modulation tailored to the distinct immunosuppressive mechanisms within the HCC TME. Collectively, these trials highlight the therapeutic potential of dual checkpoint blockade in HCC while reinforcing the importance of biomarker-guided approaches. Future strategies will benefit from integrating immune phenotyping, molecular profiling, and real-time monitoring to identify patients most likely to benefit from intensified immunotherapy regimens.

### Comparative immunological mechanisms of combination immunotherapies in HCC

The rationale behind combination immunotherapies in HCC lies in leveraging distinct yet complementary immunomodulatory mechanisms. In ICIs-TKIs combinations, TKIs not only exert antiangiogenic effects but also modulate the immune microenvironment by reducing Tregs, MDSCs, and TAMs, while enhancing cytotoxic T cell infiltration and activity. This immunomodulatory shift creates a more permissive environment for ICIs to exert their function. In contrast, ICIs-chemotherapy combinations rely on the capacity of certain chemotherapeutic agents to induce ICD, which promotes antigen release, enhances dendritic cell maturation, and facilitates T cell priming. Additionally, chemotherapy can transiently deplete immunosuppressive cells, amplifying the effectiveness of ICIs. Meanwhile, dual ICI regimens, such as PD-1/PD-L1 blockade combined with CTLA-4 inhibition, aim to overcome immune resistance by targeting non-redundant inhibitory pathways. CTLA-4 blockade enhances early-stage T cell priming and proliferation in lymphoid tissues, whereas PD-1/PD-L1 inhibition reinvigorates exhausted T cells within the tumor microenvironment. The distinct mechanisms of these strategies provide multiple avenues to reprogram the immunosuppressive environment of HCC, thereby enhancing anti-tumor immunity through synergistic interactions.

## Limitations of preclinical models in studying combination therapies

### Translational barriers: why preclinical models fail to predict clinical outcomes in HCC

Despite the clinical promise of combination immunotherapies in HCC, progress in mechanistic understanding remains hampered by the limitations of current preclinical models. Most preclinical studies focused on single-agent interventions (e.g., ICIs or TKIs alone) and failed to recapitulate the immunologic, fibrotic, and vascular complexity of the human HCC TME, limiting their predictive value for combination regimens. Moreover, multiple barriers have constrained the development of robust combination models, including scientific challenges in mechanistic validation, economic disincentives related to shared intellectual property, and regulatory requirements that demand independent proof of efficacy and safety for each component [110]. As a result, many therapeutics that show efficacy in preclinical settings ultimately fail in clinical trials.

Furthermore, the most commonly used models—particularly murine systems—do not capture the full biological context of human HCC. Key features such as liver fibrosis, chronic inflammation, and the heterogeneous immune landscape are often absent or poorly represented. Differences in hepatic drug metabolism between mice and humans further complicate translational interpretation, resulting in discrepancies in pharmacokinetics, toxicity, and immune responses. Many models also fail to simulate the long-term immune adaptations and resistance mechanisms that emerge under prolonged treatment, as most studies assess only short-term tumor control [111]. Moreover, unlike human HCC, which typically arises with a background of chronic liver disease (e.g., cirrhosis, viral infection, or MASLD), most preclinical models lack this inflammatory background [112]. Given that ICIs rely on functional T cells and antigen presentation, commonly used models (e.g., xenografts) do not adequately represent these features and are therefore poorly suited for evaluating immunotherapy. This gap highlights the need for enhanced systems that accurately reflect the co-evolution of tumor, stroma, and immune components in HCC.

### Model-specific constraints: evaluating the fidelity of preclinical systems in immunotherapy research

Despite the diversity of preclinical models used to study HCC, none fully captures the immunologic and stromal intricacies necessary to evaluate combination immunotherapy strategies. This shortfall stems not just from individual technical limitations, but from the more profound mismatch between model design and the defining pathophysiology of HCC, which is a cancer that evolves in the setting of chronic liver injury, fibrosis, and a profound immune remodeling hepatic environment.

Immunodeficient mouse models, such as patient-derived xenografts (PDXs) or cell line-derived xenografts (CDXs), are widely used due to their ability to support the growth of human tumors. However, their utility in immunotherapy research is severely constrained. These models lack a functional immune system, eliminating critical tumor–immune interactions necessary for evaluating immunomodulatory therapies [113]. While structurally human, they are immunologically inert and thus incapable of recapitulating the dynamic immune remodeling that underpins immunotherapy response or resistance. Conversely, genetically engineered mouse models (GEMMs) and syngeneic tumor models provide intact murine immunity, enabling immune profiling and evaluation of checkpoint blockade. Yet, they too fall short. GEMMs introduce key oncogenic drivers, such as *TP53* loss or *β-catenin* activation, but typically fail to recapitulate the chronic inflammation, cirrhosis, and fibrosis that define the clinical HCC context [114]. Similarly, syngeneic models, such as Hepa1-6 in C57BL/6 mice, support immune profiling; however, they lack the genomic complexity of human HCC and are typically implanted into non-fibrotic, immunologically naïve livers. Thereby, limiting translational fidelity in terms of tumor heterogeneity, TME architecture, and liver-specific immunobiology, in which critical mechanisms of immune exclusion, resistance, and fibrosis-driven immune suppression are underrepresented.

Humanized mouse models offer a partial solution by engrafting immunodeficient mice with human CD34^+^ hematopoietic stem cells, peripheral blood mononuclear cells (PBMCs), or humanized-bone marrow, liver, thymus (Hu‐BLT), enabling the evaluation of human immune-tumor interactions. However, they remain technically challenging, expensive, and time-limited due to complications like graft-versus-host disease (GVHD) [115–117]. Moreover, immune reconstitution is often incomplete, lacking important components such as tissue-resident memory T cells and fully functional antigen-presenting cells. Variability in engraftment success and short experimental windows Limit their reproducibility and translational value. Efforts to bridge these gaps have led to the use of 3D organoids and tumor-immune co-culture systems to dissect immune interactions in vitro. These reductionist models enable the controlled manipulation of immune and stromal elements; however, they lack vasculature, fibrosis, and systemic immune feedback [118]. More novel platforms are under development. A major thrust is cytokine-humanized strains, which are mice engineered to express human cytokines essential for myeloid and NK cell development, such as the MI(S)TRG model [119]. Advances in thymus sourcing also help. For example, the NeoThy model can replace scarce fetal thymus with pediatric thymic tissue from surgeries, providing ample human thymic epithelial cells for T-cell education and yielding BLT-like immunity without the need for fetal tissue [120].

A key limitation across nearly all models is the short duration of experimentation, which precludes the study of longitudinal immune remodeling, therapy-induced resistance, and chronic adaptation under immune pressure. Given that many resistance mechanisms in HCC develop gradually, such as compensatory checkpoint upregulation, stromal remodeling, or metabolic rewiring, current preclinical timelines often miss these clinically relevant dynamics. To advance combination immunotherapy research, the field must transition toward multidimensional platforms that integrate immune, stromal, vascular, and fibrotic elements within a disease-relevant hepatic context. This may involve hybrid models combining fibrosis-inducing protocols with syngeneic tumors, or orthotopic implantation into fibrotic livers. Incorporating spatial and temporal complexity, including chronic inflammation and matrix remodeling, is essential for accurately modeling immune resistance and therapeutic outcomes.

## Emerging and novel concepts in combination therapy

### Tumor heterogeneity and immune phenotypes: determinants of combination therapy response

HCC is a highly heterogeneous Malignancy, both in its origins and in the immunologic architecture of its TME. This heterogeneity underpins the differential responses observed with ICI therapy. Epidemiologically, HCC arises from diverse etiological backgrounds, including chronic HBV or HCV infection, alcohol-related Liver disease, and MaSH. Globally, HBV and HCV account for approximately 65% of HCC cases, particularly in Asia and Africa [121]. HBV is oncogenic even in the absence of cirrhosis due to its integration into host DNA, whereas HCV typically drives carcinogenesis through chronic inflammation and regeneration in cirrhotic livers [122]. In contrast, the incidence of MASH-related HCC is increasing rapidly in Western countries due to the obesity epidemic. Unlike viral HCC, MASH-HCC often develops in non-cirrhotic livers, reflecting a different immunopathogenic process characterized by metabolic stress, chronic low-grade inflammation, and gut microbiota dysbiosis. Alcohol-related HCC commonly occurs in the context of advanced fibrosis or cirrhosis and is associated with long-term oxidative stress and toxin-induced genomic damage [123, 124].

These distinct etiologies may shape the immune landscape of HCC, influencing treatment efficacy and response rates to immunotherapy. Preclinical and clinical data suggest that ICIs are more effective in viral-related HCC than in MASH-HCC. Pfister et al. showed that anti–PD-1 therapy paradoxically worsened HCC in MASH models and that MASH-HCC patients had shorter survival on PD-1/PD-L1 blockade. A meta-analysis of phase III trials found no survival benefit from ICIs in non-viral HCC, and small cohorts of MASLD-HCC patients experienced worse outcomes with immunotherapy [125]. By contrast, subgroup analysis of IMbrave150 showed longer OS in Chinese patients (high HBV prevalence) than in the global cohort, hinting that viral HCC may derive greater benefit. Notably, a meta-analysis by Ho et al. reported no significant difference in ORR between viral and non‑viral HCC treated with ICIs, so the issue remains debated[123].

On a genomic level, among the most well-studied genetic drivers of immune resistance in HCC are *CTNNB1* (β-catenin) mutations, which are present in nearly 27% of HCC cases [126], which can impair DCs'recruitment and antigen presentation, resulting in diminished CD8^+^ T cell infiltration into the tumor parenchyma [127, 128]. In parallel, gain-of-function mutations in *CTNNB1* have been associated with increased expression of Matrix metallopeptidase 9 (MMP9), which further hinders CD8⁺ T cell cytotoxic function, contributing to resistance to anti-PD-1 therapy [129].

**Fig. 6:**
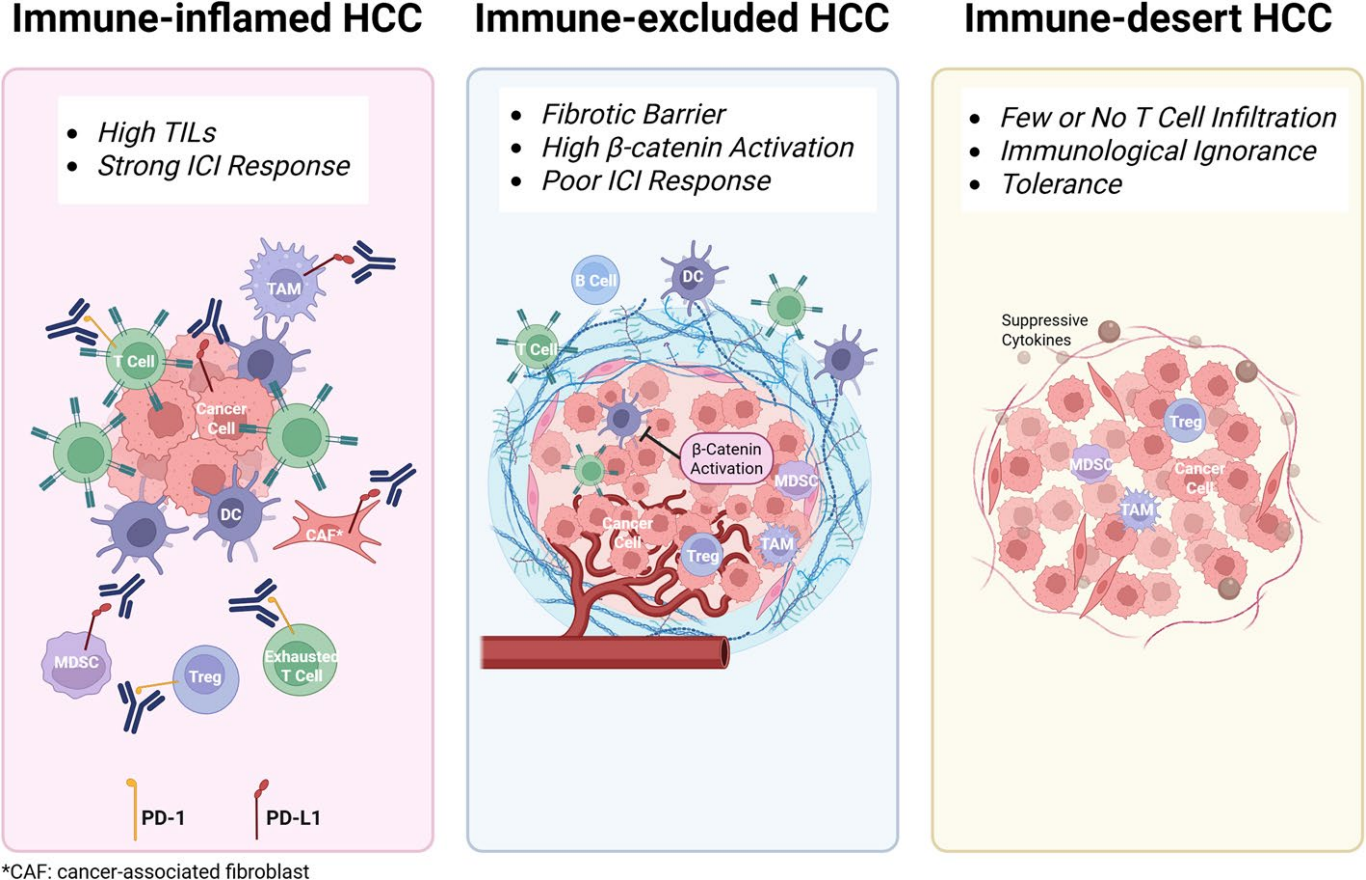
Immune phenotypes of HCC tumors and their implications for ICI response. HCC tumors can be classified into three major immune phenotypes: immune-inflamed, immune-excluded, and immune-desert. Immune-inflamed tumors are characterized by dense infiltration of CD8⁺ T cells in the tumor parenchyma and are typically more responsive to ICIs. Immune-excluded tumors exhibit abundant immune cells in the surrounding stroma but limited infiltration into tumor nests, often due to fibrotic barriers or β-catenin–mediated suppression of DC recruitment. Immune-desert tumors lack meaningful T cell infiltration altogether, reflecting impaired immune priming or antigen presentation. These phenotypic distinctions are associated with differential responses to immunotherapy, highlighting the need for stratified combination strategies in HCC

Recent frameworks based on the degree and spatial distribution of immune cell infiltration classify HCC tumors into three immunologic phenotypes, including immune-inflamed, immune-excluded, and immune-desert. Immune-inflamed tumors exhibit robust infiltration of TILs within the parenchyma and are generally more responsive to ICI-based therapies. In contrast, immune-excluded tumors show immune cell presence confined to the peritumoral stroma, often due to fibrotic barriers or β-catenin pathway-mediated disruption of DC trafficking. Immune-desert tumors, by comparison, are devoid of significant lymphocytic infiltration, reflecting more profound deficits in immune priming [128, 130, 131]. As illustrated in Fig. 6, these phenotypes reflect distinct modes of immune dysfunction, each with different implications for therapeutic response.

Clinical evidence supports the relevance of these immune profiles for treatment outcomes. Trials such as GO30140 and IMbrave150 have shown that patients with inflamed tumors, identified by high TMB or IFN-γ–associated gene expression, demonstrate significantly better responses to ICI combinations than patients with non-inflamed tumors [132, 133]. These findings suggest that immune phenotype is a key determinant of ICI efficacy in HCC and may serve as a foundation for rational treatment selection.

To capitalize on these insights, several emerging technologies, such as scRNA-seq, spatial transcriptomics, and multi-omics integration, have significantly deepened our understanding of diverse immune cell states and exhaustion profiles within tumors, as well as their therapeutic implications at the cellular level [134]. Multi-omics platforms that integrate genomic, transcriptomic, and proteomic data are being applied to identify predictive biomarkers and guide therapy matching [135]. In addition, spatial transcriptomics has introduced the ability to localize immune, stromal, and malignant cell populations within tumor tissue, allowing researchers to distinguish, for example, between exclusion caused by fibrosis versus exclusion driven by molecular signaling pathways, such as the Wnt/β-catenin pathway [136, 137]. Moreover, biomarker-guided stratification based on TME immune signatures, including TIL density and IFN-γ–associated gene expression, has shown potential in predicting responsiveness to combination immunotherapies. These tools may enable more precise, context-specific intervention strategies in the future.

### Epigenetic regulation of immune evasion and ICI + TKI therapeutic resistance

While much attention in HCC immunotherapy has focused on cellular interactions within the TME, mounting evidence suggests that epigenetic dysregulation plays a central and underappreciated role in HCC immune evasion, modulating gene expression without altering the underlying DNA sequence. Aberrant DNA methylation and histone modification are among the most prominent epigenetic mechanisms implicated in immune suppression in HCC. Hypermethylation of promoter regions and the removal of activating histone marks can silence genes essential for antigen presentation, such as major histocompatibility complex class I (MHC class I) molecules, and upregulate immunosuppressive markers, including PD-L1, and thereby reinforce a microenvironment of T cell exhaustion. Likewise, DNA methyltransferase 1 (DNMT1)-mediated methylation has been linked to the upregulation of PD-L1 in sorafenib-resistant HCC cells [138]. Histone deacetylases (HDACs), particularly HDAC2 and HDAC9, as well as DNMT, contribute to this process by removing histone acetylation marks, leading to transcriptional repression of MHC class I. On the opposing axis, histone acetyltransferases (HATs) mediate chromatin relaxation and gene activation [139, 140]. For example, HAT1 has been found to be upregulated in HCC and promotes tumor growth in HCC. Knockdown of HAT1 resulted in decreased expression of gluconeogenesis-related genes, such as FBP1, and upregulation of glycolysis-associated genes, including LDHA, GLUT1, and PKM2 [141].

Adding another layer of regulation, noncoding RNAs (ncRNAs), particularly long noncoding RNAs (lncRNAs), modulate key immunosuppressive pathways in HCC. For instance, NNT-AS1 has been shown to activate TGF-β signaling and suppress CD4⁺ T cell infiltration, thereby fostering an immunosuppressive phenotype [142]. Conversely, the loss of LINC01056 has been associated with acquired resistance to sorafenib, suggesting that ncRNA dysregulation contributes not only to baseline immune evasion but also to adaptive resistance mechanisms [143]. Thus, ncRNAs operate alongside DNA and histone modifications as epigenetic regulators with immunological consequences.

These mechanistic insights have stimulated growing interest in the therapeutic potential of epigenetic modifiers. Agents such as DNA methyltransferase inhibitors (DNMTis) and HDAC inhibitors (HDACis) are being evaluated as adjuncts to immunotherapy in HCC. Preclinical studies have shown that combined treatment with 5-aza-2'-deoxycytidine (a DNMTi) and trichostatin A (an HDACi) can reprogram M2-polarized TAMs into an M1-like, pro-inflammatory state, reducing secretion of TGF-β and IL-10 and enhancing T cell activation [144]. Another example is the selective inhibition of HDAC8, which increases histone H3 lysine 27 (H3K27) acetylation, thereby restoring chemokine production, enhancing CD8⁺ T cell infiltration, and improving the response to anti-PD-L1 therapy [145]. Similarly, the HDAC inhibitor belinostat has been found to synergize with CTLA-4 blockade, increasing IFN-γ production, reducing Treg infiltration, and restoring antigen visibility [146].

These therapeutic rationales are now being explored in early-phase clinical trials. A promising example is the ongoing study of zabadinostat (Zaba), an HDAC inhibitor, in combination with the PD-1 antibody geptanolimab (GB226) (NCT05873244). Preliminary findings suggest that this combination may help reverse ICI resistance in subsets of patients with HCC, although further validation is required. However, these agents are broad-acting and associated with significant off-target effects, particularly when combined with ICIs or TKIs. Clinical translation is still in its early-phase trials, underscoring the need for predictive biomarkers to guide patient selection. In addition to molecular markers, such as global DNA methylation or histone acetylation profiles, advanced immune phenotyping may also be helpful. For instance, more recently, Tu et al. identified HDAC1/2/3 overexpression as a feature of ICI-resistant HCC with impaired IFN-γ signalling. Using single-cell multiomics and functional studies, they demonstrated that the class I HDAC inhibitor CXD101 could re-sensitize these tumors by promoting H3K27 acetylation of IFN-γ-responsive genes, inducing CD8⁺ T cell infiltration, and STAT1-GSDME-mediated pyroptosis [147]. These findings demonstrate the potential value of integrating molecular and immune phenotyping—such as HDAC expression and T cell infiltration status—to identify patients most likely to benefit from epigenetic-based ICI strategies.

### Microbiome–immune interactions: a modifiable axis for enhancing immunotherapy

The gut microbiome has emerged as a dynamic and systemic regulator of both innate and adaptive immunity, with growing evidence linking its composition, metabolic output, and structural integrity to anti-tumor immunity. Although anatomically distinct, the gut and liver are connected via the portal circulation, forming an immunologic continuum known as the gut–liver axis. Through this axis, gut-derived microbial signals influence hepatic immune tone, making the microbiome a promising, modifiable determinant of ICI efficacy in HCC.

Patients harboring a favorable microbial composition, characterized by enrichment in beneficial taxa such as *Akkermansia muciniphila* (Akk) and *Bifidobacterium,* tend to exhibit improved responses to PD-1 blockade [148]. In a recent MAFLD-HCC mouse model, depletion of Akk led to ICI resistance, whereas supplementation with live Akk, combined with PD-1 blockade, maximally suppressed tumor growth. Mechanistically, Akk repaired gut barrier integrity, lowered systemic lipopolysaccharide (LPS) and bile acid levels, and reduced suppressive monocytic MDSCs and M2-macrophages, thereby restoring T cell activity [149].

One key mechanistic contributor to this benefit is the microbial production of short-chain fatty acids (SCFAs), including acetate, propionate, and butyrate. SCFAs enhance antitumor immunity by promoting T cell activation, reducing immunosuppressive populations such as Tregs and MDSCs, and boosting antigen presentation in the TME. Intriguingly, these metabolites also exhibit functional overlap with epigenetic therapies [84]. Acting as natural HDAC inhibitors, SCFAs signal not only to inhibit HDAC activity but also to reduce HDAC protein levels and modulate immune signaling through G protein–coupled receptors, such as GPR41 and GPR43 [150]. These effects extend beyond the gut to mediate a downstream systemic immune response, fostering a proinflammatory immune milieu that supports ICI activity.

In contrast, gut dysbiosis—arising from liver disease, diet, or antibiotic use—can disrupt immune homeostasis. Dysbiosis impairs DC function, sustains the expression of inhibitory checkpoints such as PD-1 and CTLA-4, and facilitates chronic inflammation, collectively skewing the TME toward immune suppression [151–153]. Notably, antibiotic exposure prior to ICI initiation has been associated with reduced treatment efficacy, likely due to the depletion of commensal microbes that are essential for immune priming [154]. These findings have prompted growing interest in strategies to reshape the microbiome in favor of immune activation therapeutically. Among these, fecal microbiota transplantation (FMT), which transfers stool from ICI responders to non-responders, has demonstrated the capacity to enhance immunotherapy response in both preclinical models and early-phase clinical studies [155, 156]. FMT is complemented by less invasive and more scalable interventions currently under investigation, including dietary fiber enrichment, prebiotic supplementation, and probiotic therapy, all of which aim to restore microbial diversity and support the production of immunomodulatory metabolites [157].

In parallel, the field is actively exploring microbial biomarkers that predict ICI responsiveness. Recent analyses have identified enrichment of *Lachnoclostridium*, reduced *Prevotella 9* abundance, and elevated levels of bile acids, particularly ursodeoxycholic acid (UDCA) and ursocholic acid (UCA), were observed in responders to PD-1 therapy [158]. Mechanistically, UDCA has been linked to the degradation of TGF-β, a key immunosuppressive cytokine in the HCC microenvironment, suggesting a potential synergy with PD-1 inhibitors [159]. Clinical translation is progressing through trials such as the MET4-IO trial, which investigates the use of Microbial Ecosystem Therapeutic 4 (MET4) in combination with ICIs to optimize immunotherapy response in solid tumors, including HCC [160]. Altogether, these findings position the gut microbiome as a dynamic and actionable determinant of immunotherapy success. Continued exploration of microbial composition, function, and metabolic outputs may not only uncover predictive biomarkers but also unlock novel strategies to enhance ICI efficacy in HCC.

### Beyond PD-1 and CTLA-4: emerging immune checkpoint targets in HCC

While blockade of PD-1 and CTLA-4 has marked a milestone in cancer immunotherapy, the proportion of HCC patients achieving durable responses remains limited. Resistance arises from both tumor-intrinsic factors and adaptive immune evasion, prompting investigation into alternative inhibitory pathways that may undermine or compensate for PD-1/CTLA-4 targeting.

A key challenge is the persistence of T cell exhaustion despite PD-1 inhibition. Exhausted T cells in HCC frequently co-express multiple inhibitory receptors, including LAG-3, TIM-3, T cell immunoreceptor with Ig and immunoreceptor tyrosine-based inhibitory motif (ITIM) domains (TIGIT), and V-domain Ig suppressor of T cell activation (VISTA). Although these checkpoints act through distinct molecular mechanisms, they converge on suppressing cytotoxic lymphocyte activity and reinforcing an immune-silent TME. Their compensatory expression following PD-1 blockade positions them as rational targets for combination therapy.

Among these, LAG-3 has emerged as a leading target. LAG-3 is expressed on chronically stimulated T cells, amplifies exhaustion by binding to MHC class II and inhibiting IFN-γ signaling and T cell activation. In HCC, LAG-3 expression correlates with impaired immune infiltration and poorer prognosis. Its therapeutic synergy with PD-1 blockade has been validated in other cancers [161]. Clinical evidence from the RELATIVITY-047 trial in melanoma has validated the therapeutic synergy between LAG-3 and PD-1 inhibition, as combining relatlimab (anti-LAG-3) with nivolumab improved outcomes over PD-1 monotherapy, leading to FDA approval in melanoma [162]. In HCC, this approach is being evaluated in ongoing trials such as NCT05337137, NCT04567615, and NCT06320080.

TIM-3, often co-upregulated with PD-1 in TILs, serves as another compensatory checkpoint. It promotes immune tolerance by interacting with ligands such as galectin-9 and phosphatidylserine, leading to T cell apoptosis and terminal exhaustion. TIM-3 upregulation is particularly common in tumors that have progressed after PD-1 therapy, highlighting its role in acquired resistance [163]. Ongoing trials (e.g., NCT05975645, NCT03680508) are investigating whether co-blockade with PD-1 inhibitors can rescue exhausted T cells and restore antitumor immunity in HCC.

TIGIT, another promising target, is expressed on both T cells and NK cells. By interacting with its ligand CD155, TIGIT suppresses effector function across innate and adaptive compartments [164]. Inhibitors are currently being tested in combination with PD-1/PD-L1 agents in HCC to amplify cytotoxic activity and reverse tumor-induced immune tolerance. Clinical evaluation includes trials NCT06921785, NCT05904886, NCT06349980, NCT06558227, and NCT05724563, which examine anti-TIGIT agents as part of multi-checkpoint blockade strategies in HCC.

VISTA represents a distinct axis of immune suppression. Predominantly expressed on MDSCs and Tregs, VISTA limits antigen presentation and dampens T cell activation, contributing to a suppressive myeloid niche in HCC [165]. While preclinical data support its blockade, clinical development is still in early phases, and no large-scale HCC-specific trials have yet been conducted. Nonetheless, VISTA blockade remains a promising avenue for enhancing the effects of upstream checkpoint inhibition, particularly when integrated into rational combination approaches.

Together, these alternative checkpoints highlight the limitations of monotherapy and underscore the need for rational combinations tailored to the immune profile of each tumor. Emerging strategies, including bispecific antibodies and engineered fusion proteins, are being developed to simultaneously target PD-1 alongside one or more of these secondary checkpoints while minimizing systemic toxicity. The success of such approaches will depend on biomarker-guided selection of patients, particularly those with co-expression of exhaustion markers or defined resistance phenotypes. As summarized in Table 3, multiple early-phase trials are underway to assess the safety and efficacy of these next-generation immunotherapies in HCC, intending to expand the scope and durability of immune-based treatments.

**Table 3:** Novel immunotherapy targets and current clinical trials of combination therapies in HCC

Checkpoint	Primary Ligand	Immune Cell Target	Main Mechanism of Immunosuppression	Potential Inhibitor	Company	ClinicalTrials.gov	Trial Name	Phase
LAG-3	MHC class II	Exhausted T cells	Inhibits TCR signaling	Relatlimab	Bristol Myers Squibb (BMS)	NCT05337137	RELATIVITY-106	Phase 1/2
						NCT04567615	RELATIVITY-073	Phase 2
TIM-3	Galectin-9	CD8 + T cells, Tregs	Induces T cell exhaustion and apoptosis	Cobolimab	GlaxoSmithKline (GSK)	NCT03680508		Phase 2
				TQB2618	Chia Tai Tianqing	NCT05975645		Phase 1b
TIGIT	CD155	T cells, NK cells	Competes with CD226 to suppress T and NK cell cytotoxicity	Tiragolumab	Genentech/Roche	NCT05904886	SKYSCRAPER-14	Phase 3
				Rilvegostomig	AstraZeneca	NCT06921785	ARTEMIDE-HCC01	Phase 3
				domvanalimab	Arcus Biosciences	NCT05724563		Phase 3
VISTA	PSGL-1 (at acidic pH)	MDSCs	Inhibits T cell activation and proliferation	KVA12123	Kineta	NCT05708950	VISTA-101	Phase 1/2
**ClinicalTrials.gov**	**Trial Name**	**Indications**	**Combination Therapy**	**Targets**	**Phase**	**Company**	**Country/Region**	**Condition**
NCT06320080		Advanced HCC	TQB2223 + penpulimab	LAG-3 + PD-1	Phase 1b	Chia Tai Tianqing	China	Recruiting
NCT05337137	RELATIVITY-106	Untreated advanced/metastatic HCC	relatlimab + nivolumab + bevacizumab	LAG-3 + PD-1 + VEGF	Phase 1/2	Bristol Myers Squibb (BMS)	Multi-region	Active, not recruiting
NCT04567615	RELATIVITY-073	Patients with advanced liver cancer who have never been treated with immuno-oncology therapy, after prior treatment with TKIs	relatlimab + nivolumab	LAG-3 + PD-1	Phase 2	Bristol Myers Squibb (BMS)	Multi-region	Active, not recruiting
NCT05975645		Advanced HCC	TQB2618 + penpulimab + anlotinib	TIM-3 + PD-1 + multitarget TKI	Phase 1b	Chia Tai Tianqing	China	Recruiting
NCT03680508		Locally advanced or metastatic liver cancer	cobolimab + dostarlimab	TIM-3 + PD-1	Phase 2	GlaxoSmithKline (GSK)	America	Active, not recruiting
NCT06349980		Unresectable, locally advanced or metastatic HCC	HLX53 + serplulimab + HLX04	TIGIT + PD-1 + VEGF	Phase 2	Shanghai Henlius Biotech	China	Recruiting
NCT06558227		Advanced HCC	ZG005 + bevacizumab	TIGIT + VEGF	Phase 2	Suzhou Zelgen Biopharmaceuticals	China	Recruiting
NCT06921785	ARTEMIDE-HCC01	Advanced HCC who are not amenable to curative therapy or locoregional therapy	rilvegostomig + bevacizumab (+ tremelimumab)	TIGIT × PD-1 + VEGF (+ CTLA-4)	Phase 3	AstraZeneca	Multi-region	Not yet recruiting
NCT05904886	SKYSCRAPER-14	Unresectable, locally advanced or metastatic HCC	tiragolumab + atezolizumab + bevacizumab	TIGIT + PD-L1 + VEGF	Phase 3	Roche	Multi-region	Active, not recruiting
NCT05724563		Advanced liver and bile duct cancers	domvanalimab + zimberelimab	TIGIT + PD-1	Phase 3	Arcus Biosciences	America	Recruiting

### Stromal and fibrotic barriers: modulating the TME to improve ICI efficacy

The fibrotic TME of HCC presents a formidable obstacle to immune infiltration and therapy efficacy. Unlike many tumors that arise in immunologically naïve tissue, HCC is embedded in a microenvironment dominated by activated hepatic stellate cells (HSCs), dense extracellular matrix (ECM), and immunosuppressive stromal components. This desmoplastic architecture limits immune cell access and simultaneously fosters immunosuppressive signaling cascades that attenuate the effects of immunotherapy. Key mediators include TGF-β, the CXCR4–CXCL12 chemokine axis, and lysyl oxidase-like 2 (LOXL2), which contribute to the immunosuppressive architecture of HCC.

TGF-β, secreted by Tregs, MDSCs, and TAMs, plays a dual role in driving hepatic fibrosis and suppressing cytotoxic immune responses. Through the activation of HSCs, TGF-β promotes ECM deposition and structural remodeling. Simultaneously, it impairs CD8⁺ T cell function and expands Treg populations, thereby tipping the balance of the TME toward immune evasion [166, 167]. In preclinical models, inhibition of TGF-β signaling has been shown to re-enable T cell infiltration and sensitize tumors to PD-1/PD-L1 blockade. For example, the TGF-β receptor I kinase inhibitor galunisertib, when combined with anti-PD-L1 therapy, resulted in significant tumor regression and enhanced T cell accumulation in murine HCC models [168].

Another critical pathway implicated in immune exclusion is the CXCR4–CXCL12 axis. C-X-C chemokine receptor type 4 (CXCR4), a chemokine receptor expressed on Tregs and MDSCs for chemokine (C-X-C motif) Ligand 12 (CXCL12), mediates their recruitment into the tumor bed. Its blockade (e.g., plerixafor) has been shown to reverse immune suppression and potentiate ICI efficacy [169]. In murine models, combination therapy with CXCR4 antagonists and PD-1 blockade not only suppressed Tumor growth and prolonged survival but also reprogrammed intratumoral conventional type 1 DCs (cDC1s) with increased abundance and heightened functional activity, thereby enhancing CD8⁺ T cell priming and infiltration and restoring sensitivity to ICIs[170].

Additionally, LOXL2, produced mostly by activated HSCs and myofibroblasts, crosslinks collagen and contributes to ECM stiffening, attenuating the fibrotic matrix-induced shield against immune attack. LOXL2 expression is upregulated under hypoxic conditions through HIF-1α signaling, and its activity has been associated with tumor progression and immune exclusion in HCC [171, 172]. Preclinical studies suggest that LOXL2 inhibition may soften the fibrotic scaffold by reducing matrix density and facilitating immune cell infiltration into tumor nests, although clinical translation remains in its early stages [173].

As combination immunotherapy evolves, strategies that disrupt stromal architecture and reduce myeloid suppression may prove essential for converting immunologically “cold” tumors into treatment-responsive ones. Moreover, the combination targeting fibrotic and myeloid compartments of the TME offers a promising complement to existing immunotherapies, yet warrants the use of orthotopic and fibrosis-inducing murine models to better evaluate combination strategies in a context that mirrors the complex pathology of human HCC. Despite these advances, targeting the stroma presents challenges. Stromal components also contribute to tissue repair and homeostasis, and indiscriminate inhibition may result in hepatotoxicity or impaired liver regeneration. Thus, future trials will need to prioritize biomarker-driven patient selection and mechanistic endpoints to determine which elements of the fibrotic microenvironment are therapeutically targetable, thereby enhancing immune engagement in HCC.

## Future perspectives and unanswered questions

### Biomarker-driven combination therapy

Despite the growing momentum in combination immunotherapies in HCC, their clinical efficacy remains limited to a subset of patients. This variability underscores a central unmet need for the identification of predictive biomarkers that can stratify responders, anticipate resistance, and guide rational treatment selection. While considerable progress has been made in other cancers, biomarker development in HCC has fallen behind due to tumor complexity, variable etiologies, and the immunologically unique hepatic environment.

Traditional biomarkers such as PD-L1 expression have shown limited predictive value in HCC. Expression patterns are heterogeneous, which vary across tumor regions, immune compartments, and treatment phases, and the correlation between PD-L1 levels and ICI response remains inconsistent. Similarly, TMB, while useful in some malignancies, is generally low in HCC. A large cohort study (N = 755) reveals low median TMB values (approximately 4 mutations/Mb), with < 1% of patients exhibiting high TMB (≥ 20 mutations/Mb), reducing its standalone predictive value [174]. Other studies with small HCC samples (N < 50) also support the low TMB (< 6 mutations/Mb) condition at the median level [175, 176]. Nevertheless, TMB may retain prognostic significance when incorporated into multiplex models that integrate immune activation signatures or the quality of neoantigens.

In contrast, features of the immune contexture, particularly in the presence and localization of TILs, offer more consistent predictive value. High densities of CD8⁺ effector T cells in TILs are associated with improved ORRs of ICI-based therapies, more than doubling compared to TIL-low counterparts and superior survival outcomes in patients [177]. However, the spatial distribution of these TILs in immune-inflamed tumors is responsive to ICIs. Specifically, in cases where T cells infiltrate the tumor core, they respond more favorably than immune-excluded or immune-desert phenotypes. This highlights the need for spatially resolved profiling approaches. Technologies such as spatial transcriptomics and multiplexed immunohistochemistry are increasingly being utilized to distinguish immune activation patterns, characterize fibrotic barriers, and reveal exclusion mechanisms, including β-catenin pathway activation or ECM remodeling, that impact therapeutic efficacy.

Beyond the tumor itself, systemic and host-related biomarkers are emerging as important contributors to immunotherapy outcomes. The gut microbiome, connected to the liver via the portal circulation, plays a key role in shaping immune status through microbial metabolites, bile acid modulation, and barrier integrity. High enrichment of beneficial commensal bacteria, such as *Akkermansia muciniphila,* *Ruminococcaceae*, and *Lachnospiraceae*, correlates with enhanced cytotoxic T cell infiltration and improved ICI response, likely through enhanced antigen presentation, reduced Treg/MDSC burden, and favorable cytokine modulation. Conversely, dysbiotic profiles, dominated by potentially pathogenic bacteria such as *Proteobacteria*, are associated with immunosuppressive signaling and treatment resistance [178]. Similarly to dysbiosis, antibiotic exposure has been linked to poor outcomes. Microbial metabolites such as SCFAs and bile acids, particularly UDCA, may serve as mechanistically relevant biomarkers and potential therapeutic targets.

Beyond static biomarkers, dynamic biomarkers, such as circulating tumor DNA (ctDNA), serum cytokine profiles, and peripheral immune cell phenotyping, offer additional value by enabling real-time monitoring of treatment response and immune adaptation. Moreover, characterization of the TME, including IFN-γ-related gene signatures, MDSC burden, and fibrosis-associated mediators such as TGF-β and LOXL2, can reveal resistance-prone niches and inform the tailoring of therapeutic approaches. In parallel, liquid biopsies, particularly, offer a non-invasive means of capturing tumor evolution and detecting early signs of resistance or response failure. These tools can complement tissue-based profiling and may eventually support longitudinal, adaptive treatment strategies.

Taken together, these advances signal a necessary shift from single, static markers toward integrated, multidimensional biomarker frameworks. Effective precision immunotherapy in HCC will require combining tumor-intrinsic features (e.g., mutation signatures, immune checkpoint expression), microenvironmental factors (e.g., TIL localization, fibrosis), and systemic cues (e.g., microbiome composition, circulating immune signals). While implementation remains constrained by technical complexity, cost, and accessibility, especially for spatial and multi-omic platforms, ongoing clinical efforts are beginning to integrate these insights into trial designs. Looking ahead, biomarker-guided treatment selection will be critical not only for maximizing initial response but also for anticipating and overcoming resistance. As discussed in the next section, the evolving immunologic landscape of HCC requires a flexible and individualized approach, one that tracks immune adaptation over time and aligns therapy with the tumor's changing vulnerabilities.

### Overcoming resistance to combination therapy

Despite the expansion of ICI-based combination therapies in HCC, resistance remains a common and often inevitable clinical challenge. Many patients exhibit either primary resistance, failing to respond from the outset, or acquired resistance after an initial response. These resistance patterns arise from a dynamic interplay of tumor-intrinsic factors, immune adaptation, stromal remodeling, and metabolic reprogramming, each posing distinct barriers to sustained therapeutic efficacy.

At the immune level, tumors often adapt to PD-1 blockade by upregulating alternative inhibitory checkpoints such as CTLA-4, LAG-3, TIM-3, or TIGIT. This compensatory upregulation sustains T cell dysfunction despite initial reactivation [179]. Clinical strategies to counter this include dual or triple checkpoint blockade, with several bispecific antibodies and multi-agent regimens currently in early-phase trials. For example, PD-1 + LAG-3 co-targeting has shown clinical promise in other cancers and is now being tested in HCC. These approaches may be particularly valuable for patients whose tumors display co-expression of exhaustion markers or who experience disease progression after monotherapy.

Resistance is also observed in tumors treated with VEGF-TKIs. While TKIs can remodel the tumor vasculature and promote immune infiltration, therapeutic pressure may lead tumors to activate alternative pro-angiogenic pathways, such as the FGF and HGF/MET signaling axes [180]. These compensatory mechanisms restore aberrant vascularization and facilitate immune escape. At the same time, VEGF pathway inhibition can induce recruitment of MDSCs and TAMs, further reinforcing the immunosuppressive TME and dampening antitumor immune responses.

In addition, resistance may stem from the TME, particularly in immune-excluded tumors, where fibrotic barriers and stromal signaling prevent T cell infiltration. Key mediators include TGF-β, the CXCR4-CXCL12 axis, and LOXL2, all of which promote immune suppression and extracellular matrix remodeling. Agents targeting these pathways, such as TGF-β receptor inhibitors (e.g., galunisertib) and CXCR4 antagonists (e.g., plerixafor), are under active investigation. Early preclinical and clinical studies suggest that these agents may reprogram the TME to restore sensitivity to immune checkpoint blockade [170].

Metabolic reprogramming provides another layer of immune evasion. In response to therapeutic stress, HCC cells may shift toward oxidative phosphorylation (OXPHOS), glutamine metabolism, or lipid utilization [181]. These adaptations support tumor survival, reduce immunogenicity, and impair T cell function by competing for key nutrients within the TME. Although most data in this area remain preclinical, combining ICIs with metabolic inhibitors may help overcome this form of resistance, particularly in metabolically active or hypoxic tumors.

Epigenetic dysregulation is also implicated. Aberrant DNA methylation and histone modifications can silence genes involved in antigen presentation, enhance PD-L1 expression, or preserve immunosuppressive phenotypes in myeloid cells. Epigenetic therapies such as HDAC inhibitors and DNMT inhibitors are being evaluated in combination with ICIs in early-phase trials and may help restore immune visibility and effector function.

Importantly, these resistance mechanisms are not uniform across patients. They vary according to tumor subtype, immune phenotype, underlying liver disease, and prior treatment exposure. For clinicians, this underscores the need for real-time profiling, including tumor sequencing, immune phenotyping, and circulating biomarkers, to monitor disease evolution and guide adaptive treatment strategies to sustain durable responses and improve long-term outcomes in this complex and heterogeneous disease.

### AI and systems biology for response prediction

The complexity and heterogeneity of HCC pose significant challenges to predicting immunotherapy outcomes using conventional clinical or molecular markers. To address this, artificial intelligence (AI) and systems biology are emerging as powerful tools for integrating high-dimensional data and generating clinically actionable insights.

AI models, particularly those based on machine learning and deep learning algorithms, can uncover patterns across diverse data modalities, radiologic imaging, serum biomarkers, multi-omic profiles, and histopathology that are difficult to discern manually. For instance, convolutional neural networks (CNNs) have been trained to analyze CT and MRI scans to non-invasively infer immune phenotypes, vascular patterns, and TME composition in HCC patients [182]. When paired with clinical covariates such as the Child–Pugh score, these models can support risk stratification and therapeutic decision-making. In parallel, systems biology approaches, using network modeling, pathway inference, and data integration, can elucidate the mechanistic underpinnings of immunotherapy response and resistance. Multi-omics platforms that combine genomics, transcriptomics, proteomics, and epigenomics enable a holistic view of tumor biology and have been applied to characterize immune-inflamed versus immune-excluded phenotypes in HCC [135]. These insights not only support patient stratification but also highlight novel therapeutic targets within immune, metabolic, and stromal pathways.

Recent advances in single-cell and spatial transcriptomics have further refined our understanding of immune–stromal crosstalk in HCC. These technologies allow for precise localization and characterization of immune cells, cancer-associated fibroblasts (CAFs), and endothelial cells within the TME. For example, spatial analysis has identified Tumor-derived secreted phosphoprotein 1 (SPP1) as a key inducer of HSCs differentiation into CAFs, promoting collagen deposition and fibrotic immune exclusion that contribute to resistance [183]. Liquid biomarkers, such as cell-free DNA (cfDNA) fragmentation patterns, ctDNA, and serum proteins like alpha-fetoprotein (AFP), can also be incorporated into AI models for real-time monitoring of tumor evolution and immunotherapy response. When integrated with multi-modal datasets, these circulating markers offer a minimally invasive route to longitudinal disease surveillance and dynamic treatment adjustment.

Despite their promise, the clinical implementation of AI and systems biology remains constrained by practical challenges, including limited availability of high-quality annotated datasets, lack of standardization across platforms, and the need for interpretable models that can guide physician decision-making. Rigorous clinical validation, transparent model architecture, and harmonized pipelines will be essential to enabling the safe and effective translation of findings into routine practice. Ultimately, integrating predictive modeling with biologically interpretable frameworks will be key to shifting HCC treatment from a reactive to an adaptive approach. As tools like spatial profiling and multi-omic machine learning mature, they may form the foundation of precision immunotherapy in HCC, enabling the matching of the proper treatment to the right patient at the right time.

### Unanswered questions and discussion

Despite the expansion of clinical trials and extensive preclinical rationale, the translational success rate for combination immunotherapy in HCC remains disappointingly low. Durable clinical benefit has been achieved in only a subset of patients, underscoring persistent obstacles in model fidelity, trial design, and patient heterogeneity. A key limitation lies in the reliance on preclinical systems that fail to replicate the fibrotic, immunosuppressive, and metabolically adapted environment of human HCC. Immunodeficient xenografts and syngeneic mouse models, while widely used, lack chronic liver inflammation, cirrhosis, and stromal complexity. As a result, combination therapies that appear efficacious under controlled experimental conditions often fail to deliver clinical benefit. Emerging fibrosis-inducing orthotopic models, humanized immune platforms, and spatial co-culture systems may offer more predictive insights and should be prioritized in translational pipelines.

The trial design itself has also contributed to clinical failures. The combination of cabozantinib and atezolizumab, for instance, demonstrated encouraging preclinical synergy but failed to significantly improve OS in the COSMIC-312 trial. This outcome highlights a critical issue: accelerated transitions from Phase I to Phase III, without adequate dose optimization, sequencing assessment, or toxicity profiling, can undermine even well-rationalized regimens and lead to costly failures [184]. Another example is the LEAP-002 trial [59], although it shares similar primary endpoints with the successful CARES-310 study [57], differences in trial design may partially explain the LEAP-002's ultimate failure. First, compared to the CARES-310, the LEAP-002 employs stricter inclusion/exclusion criteria, excluding high-risk populations such as those with Vp4 portal vein invasion and esophageal/gastric varices, thereby enrolling a higher proportion of low-risk patients. This might reduce the measurable impact of therapeutic interventions. Then the CARES-310 uses sorafenib as the control arm, whereas the LEAP-002's control group is lenvatinib plus placebo. Although the lenvatinib-plus-placebo design is optimal for the LEAP-002 trial, one might question whether replacing the control with sorafenib would have yielded different results. This hypothesis stems from the REFLECT study (NCT01761266), which demonstrates lenvatinib's superior overall therapeutic efficacy compared to sorafenib: median OS is 13.6 months vs. 12.3 months; median PFS is 7.4 months vs. 3.7 months; and ORR is 24.1% vs. 9.2%.[185]. At the same time, heterogeneity in HCC etiology, including viral status, background cirrhosis, metabolic comorbidities, and ethnic variation, further complicates the development of immunotherapy. In late-stage HCC treated with ICIs, patient etiology (e.g., HBV infection) and geographic factors (e.g., Asian populations) correlate with enhanced therapeutic efficacy. Notably, LEAP-002 and CARES-310 differ significantly in HBV-positive subgroups and Asian enrollment: 49% vs. 76% (HBV +) and 31% (excluding Japan) vs. 83% (Asian populations), respectively. These disparities likely influenced the divergent outcomes. Consequently, reliable biomarkers for patient stratification remain an urgent unmet need.

Yet even among proposed biomarkers, inconsistencies are typical. TMB, although predictive in other cancers, is rare in HCC and is prone to inter-assay variability and sampling bias. Intriguingly, TMB appears more frequently in Chinese cohorts than in Western ones (> 20 mutations/Mb: 9.3% vs. 1.0%), yet the clinical implications of this divergence remain uncertain [186]. Moreover, preanalytical variables, including tissue preservation, sequencing platform, and mutation calling pipelines, can significantly alter TMB estimates [187].

PD-L1 expression, despite its intuitive appeal, has failed to predict response in HCC consistently. Discrepancies across studies may reflect underlying biological heterogeneity, but also highlight the challenges posed by variable detection thresholds, assay platforms, and temporal dynamics. These findings underscore a broader point: single-marker strategies are unlikely to support effective clinical decision-making in the context of a highly dynamic and immunologically complex disease.

To move forward, more predictive and representative modeling platforms are needed. These should capture the inflammatory, fibrotic, and immune-excluded features of HCC. At the same time, biomarker strategies must evolve toward multiplexed frameworks that integrate spatial profiling, immune cell dynamics, genetic drivers, and metabolic context. The future of HCC immunotherapy lies in tailoring combination regimens to the evolving tumor–immune ecosystem, which is mapped not only at baseline but also dynamically across the treatment course.

## Conclusions

Combination immunotherapy has emerged as a promising strategy for advanced HCC, but its success hinges on more than the empirical pairing of agents. This review highlights the need for mechanism-based design, grounded in the immunologic, fibrotic, and metabolic architecture of HCC, to guide the development of rational, durable regimens. Moving beyond one-size-fits-all approaches will require aligning therapeutic combinations with tumor–immune phenotypes, informed by real-time profiling and dynamic biomarkers. The integration of systems biology, spatial analytics, and machine learning has the potential to transform the selection and management of treatment resistance. To alter the clinical trajectory of HCC, future research must bridge the gap between mechanistic insight and therapeutic execution, bringing precision immunotherapy from concept to practice.

## Data Availability

No datasets were generated or analysed during the current study.

## Abbreviations

AFP: Alpha-fetoprotein
AI: Artificial intelligence
Akk: Akkermansia muciniphila
APC: Antigen-presenting cell
ARG1: Arginase 1
ATP: Adenosine triphosphate
AXL: Anexelekto
CAF: Cancer-associated fibroblast
cDC1: Conventional type 1 DC
CDX: Cell line-derived xenograft
cfDNA: Cell-free DNA
CNN: Convolutional neural network
ctDNA: Circulating tumor DNA
CTLA-4: Cytotoxic T-lymphocyte-associated protein 4
CTX: Cyclophosphamide
CXCL12: Chemokine (C-X-C motif) ligand 12
CXCR4: C-X-C chemokine receptor type 4
DAMP: Danger-associated molecular pattern
DC: Dendritic cell
DNMT: DNA methyltransferase
DNMTi: DNA methyltransferase inhibitor
DOR: Duration of response
ECM: Extracellular matrix
EGFR: Epidermal growth factor receptor
FMT: Fecal microbiota transplantation
GEMM: Genetically engineered mouse model
GVHD: Graft‐versus‐host disease
H3K27: Histone H3 lysine 27
HAIC: Hepatic artery infusion chemotherapy
HAT: Histone acetyltransferase
HBV: Hepatitis B virus
HCC: Hepatocellular carcinoma
HCV: Hepatitis C virus
HDAC: Histone deacetylase
HDACi: HDAC inhibitor
HIF-1α: Hypoxia inducible factor 1-alpha
HMGB1: High mobility group box 1 protein
HSC: Hepatic stellate cell
Hu-BLT: Humanized-bone marrow, liver, thymus
ICD: Immunogenic cell death
ICI: Immune checkpoint inhibitor
IFN-γ: Interferon-gamma
IL-10: Interleukin-10
iNOS: Inducible nitric oxide synthase
irAE: Immune-related adverse event
ITIM: Immunoreceptor tyrosine-based inhibitory motif
LAG-3: Lymphocyte activation gene-3
lncRNA: Long noncoding RNA
LOXL2: Lysyl oxidase-like 2
LPS: Lipopolysaccharide
mAb: Monoclonal antibody
MASH: Metabolic dysfunction-associated steatohepatitis
MASLD: Metabolic dysfunction-associated steatotic liver disease
MDSC: Myeloid-derived suppressor cell
MET: Mesenchymal-epithelial transition factor
MET4: Microbial Ecosystem Therapeutic 4
MHC class I: Major histocompatibility complex class I
MMP9: Matrix metallopeptidase 9
NAFLD: Non-alcoholic fatty liver disease
NASH: Non-alcoholic steatohepatitis
ncRNA: Noncoding RNA
NE: Not estimable
NK cell: Natural killer cell
NMPA: National Medical Products Administration
NR: Not reached
NSCLC: Non-small-cell lung cancer
ORR: Objective response rate
OS: Overall survival
OXPHOS: Oxidative phosphorylation
PD-1: Programmed cell death protein-1
PD-L1: Programmed cell death-ligand 1
PDX: Patient-derived xenograft
PFS: Progression-free survival
PTX: Paclitaxel
RNS: Reactive nitrogen species
ROS: Reactive oxygen species
SCFA: Short-chain fatty acid
scRNA-seq: Single-cell RNA sequencing
SPP1: Secreted phosphoprotein 1
TAM: Tumor-associated macrophage
TCF-1: T cell factor-1
TCR: T cell receptor
TGF-β: Transforming growth factor-beta
TIGIT: T cell immunoreceptor with Ig and ITIM domains
TIL: Tumor-infiltrating lymphocyte
TIM-3: T cell immunoglobulin domain and mucin domain-3
TKI: Tyrosine kinase inhibitor
TMB: Tumor mutational burden
TME: Tumor microenvironment
TNF-α: Tumor necrosis factor-alpha
TRAE: Treatment-related adverse event
Treg: Regulatory T cell
UCA: Ursocholic acid
UDCA: Ursodeoxycholic acid
VEGF: Vascular endothelial growth factor
VISTA: V-domain Ig suppressor of T cell activation
